# A Drive to Driven Model of Mapping Intraspecific Interaction Networks

**DOI:** 10.1016/j.isci.2019.11.002

**Published:** 2019-11-06

**Authors:** Libo Jiang, Jian Xu, Mengmeng Sang, Yan Zhang, Meixia Ye, Hanyuan Zhang, Biyin Wu, Youxiu Zhu, Peng Xu, Ruyu Tai, Zixia Zhao, Yanliang Jiang, Chuanju Dong, Lidan Sun, Christopher H. Griffin, Claudia Gragnoli, Rongling Wu

**Affiliations:** 1 Beijing Advanced Innovation Center for Tree Breeding by Molecular Design, Center for Computational Biology, College of Biological Sciences and Technology, Beijing Forestry University, Beijing 100083, China; 2 Key Laboratory of Aquatic Genomics, Ministry of Agriculture, CAFS Key Laboratory of Aquatic Genomics and Beijing Key Laboratory of Fishery Biotechnology, Chinese Academy of Fishery Sciences, Beijing 100141, China; 3 Fujian Collaborative Innovation Center for Exploitation and Utilization of Marine Biological Resources, Xiamen University, Xiamen, Fujian 361102, China; 4 College of Fishery, Henan Normal University, Xinxiang, Henan 453007, China; 5 Applied Research Laboratory, The Pennsylvania State University, University Park, PA 16802, USA; 6 Division of Endocrinology, Diabetes, and Metabolic Disease, Translational Medicine, Department of Medicine, Sidney Kimmel Medical College, Thomas Jefferson University, Philadelphia, PA 19106, USA; 7 Molecular Biology Laboratory, Bios Biotech Multi Diagnostic Health Center, Rome 00197, Italy; 8 Center for Statistical Genetics, The Pennsylvania State University, Hershey, PA 17033, USA

**Keywords:** Biological Sciences, Evolutionary Ecology, Mathematical Biosciences

## Abstract

Community ecology theory suggests that an individual's phenotype is determined by the phenotypes of its coexisting members to the extent at which this process can shape community evolution. Here, we develop a mapping theory to identify interaction quantitative trait loci (QTL) governing inter-individual dependence. We mathematically formulate the decision-making strategy of interacting individuals. We integrate these mathematical descriptors into a statistical procedure, enabling the joint characterization of how QTL drive the strengths of ecological interactions and how the genetic architecture of QTL is driven by ecological networks. In three fish full-sib mapping experiments, we identify a set of genome-wide QTL that control a range of societal behaviors, including mutualism, altruism, aggression, and antagonism, and find that these intraspecific interactions increase the genetic variation of body mass by about 50%. We showcase how the interaction QTL can be used as editors to reconstruct and engineer new social networks for ecological communities.

## Highlights

• We develop a new theory for complex-trait mapping by integrating behavioral ecology
• This theory can characterize how QTL drive cooperation or competition in populations
• It can also illustrate how the activation of QTL is driven by ecological interactions
• The new theory leverages interdisciplinary studies of genetics, ecology, and evolution

## Graphical Abstract

**Figure:**
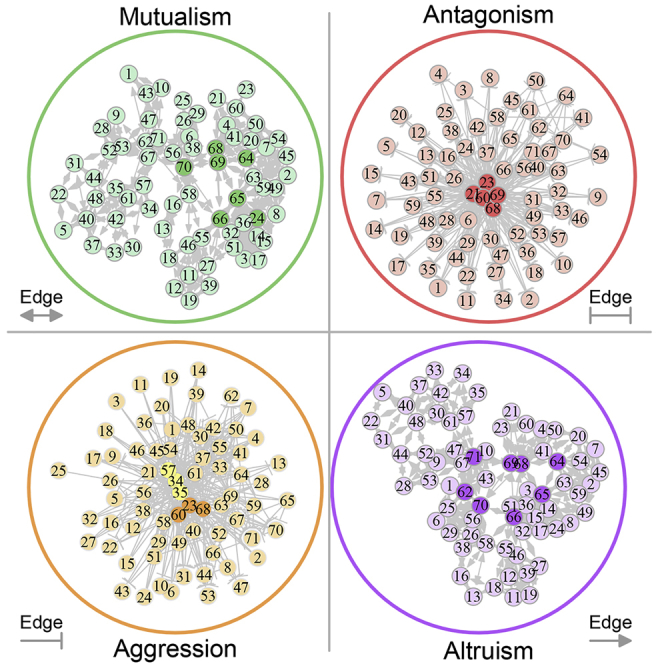

## Introduction

Quantitative genetic theory has long focused on modeling how the phenotype of an individual is determined by its genes, known as quantitative trait loci (QTL), and the environment where it grows (Ritchie et al., 2015). An increasing body of evidence has revealed that an individual's phenotype in a population is also affected by the phenotypes of other members that coexist with it (Magnuson, 1962, Wolf et al., 1998, Shuster et al., 2006, Ribas et al., 2017, Schneider et al., 2017, Santostefano et al., 2017). As such, how a particular individual performs is influenced not only directly by its own QTL, but also indirectly by the QTL of its conspecifics (Jiang et al., 2018). For instance, in an association study of laying hens, a set of genes from a single hen were identified within the serotonin pathway to affect the feather condition of its cage mates (Biscarini et al., 2010). The flowering gene FRIGIDA from focal plants in *Arabidopsis* affects the developmental processes of their neighbors, according to genetic mapping using structural equation models (Wolf et al., 2011). In *Drosophila melanogaster*, several QTL detected for aggressive behavior are at play by interacting with social environments (Rohde et al., 2017).

Although inter-individual interdependence and interactions inducing phenotypic variation involve a genetic component, existing genetic mapping theory does not enable the detailed characterization of how the underlying QTL act in a mapping population. The genetic effects of QTL may be activated by ecological interactions, such as competition, where one individual grows at the cost of others exploiting the same resources, or cooperation, by which multiple individuals can better buffer against environmental perturbations than any single one alone (Fisher and Mcadam, 2017). These ecologically meaningful QTL can be better identified if we equip a mapping approach with the ecological and social principles that can explain why an individual chooses to compete or cooperate with others. The motivation of this study is to upgrade quantitative genetic theory by embedding fundamental principles of competition and cooperation, to a level at which geneticists can map specific QTL responsible for ecological interactions, estimate how these QTL affect population phenotypes through direct and indirect effects, and test how ecological interactions can induce new genetic variation for complex traits.

To test this theory, we designed and conducted a QTL mapping experiment by genotyping a full-sib family (H1) of the common carp (*Cyprinus carpio*) and culturing its n = 71 siblings in a shared water pool. Previous cultural experiments showed that fish growth, behavior, and survival are highly plastic to the crowdedness of the environment (Magnuson, 1962, Fox and Flowers, 1990, Szkudlarek and Zakes, 2007, Ribas et al., 2017). In a natural ecosystem of coral reef, fish make their decisions to feed on algae or escape from predators according to actions of other fish (Gil and Hein, 2017). As such, we anticipate that pervasive social interactions occur among the co-cultured fish, which exert an impact on fish phenotype. Traditional mapping approaches simply associate phenotype with genotype, without considering social interactions. The application of these approaches to our mapping population detected no QTL responsible for fish body mass (Figure S1), a trait that is sensitive to competition (Magnuson, 1962). However, when the same data were analyzed under our theory, a number of QTL have been identified. To validate these discoveries, we conducted two additional mapping experiments, from each of which consistent results are obtained.

## Results

### Mathematical Descriptors of Ecological Interactions

In a socialized environment, a fish may maneuver its living territory by continuously changing its neighbors to which it pays attention (Jiang et al., 2017) so as to maximize its chance for survival and reproductive success (McFarland, 1977, Dugatkin and Reeve, 2000). This process, often guided by rational choice-based game theory (Harp, 2017), as recognized in humans (Park et al., 2017), rodents (Dias-Ferreira et al., 2009, Friedman et al., 2017), and microbes (Damore and Gore, 2012), incurs a so-called collective motion phenomenon, ubiquitous across the animal kingdom (Vicsek and Zafeiris, 2012, Jiang et al., 2017). Under natural selection, animal collective behavior has been shaped toward two tendencies. First, animals tend to swarm, flock, or shoal with individuals that resemble themselves in a cooperative way by which the so-called oddity effect, i.e., those individuals displaying difference in appearance from the group are at a greater risk to be predated (Hoare et al., 2000), can be avoided. Thus, animals of roughly similar size, color, and even smell in a population enjoy mutual cooperation and coordination (Camazine et al., 2001, Sumpter, 2006, Sumpter, 2010, Herbert-Read et al., 2011), and the similarity of two animals is proportional to the degree of the desire by which they cooperate. In mathematics, the similarity of two variables is positively correlated with their product, given that their sum is fixed. Taken together, we hypothesize that the product of two animals' body sizes can serve as a proxy for the strength of *mutualism*. In contrast, we use the inverse of the product of body sizes of two animals to approximately measure the strength of their antagonism.

**Figure 1:**
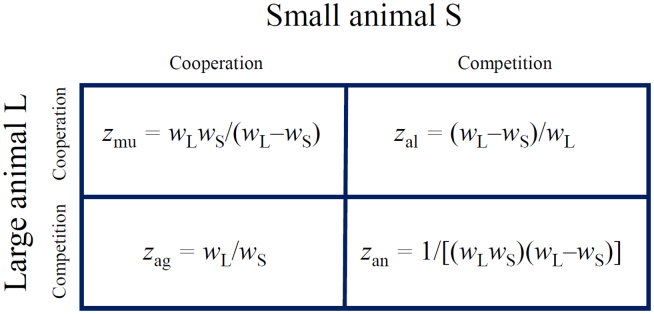
Mathematical Descriptors of Four Types of Ecological Interactions, Mutualism (*z*_mu_), Antagonism (*z*_an_), Aggression (*z*_ag_), and Altruism (*z*_al_) We use *w*_L_ and *w*_S_ to denote phenotypic values of a larger animal L and a smaller animal S, respectively, constituting a pair in a mapping population. The product of phenotypic values between two animals is used as a descriptor for the strength of mutualism, i.e., how much the two animals benefit from one another through cooperation (Zhu et al., 2016). The strength of antagonism is described by the inverse of the product of phenotypic values, reflecting how much one animal grew reciprocally at a cost of the other. To adjust the scale effect, these two descriptors are normalized by dividing them by the phenotypic difference of the larger from the smaller animal. The ratio of phenotypic values of the larger over the smaller animal is used to measure the strength of aggression, by which the former grows by harming the latter. The strength of altruism is calculated as one minus the ratio of phenotypic values of the smaller over the larger animal.

Second, animals of larger body size tend to display agonistic behavior to those of smaller body size when a limited amount of resource needs to be allocated among members of the same population (Chance and Larson, 1976, Desjardins et al., 2012, Romenskyy et al., 2017). As an aggressive and defensive action, this behavior is adaptive, widely believed to play an important role in resource acquisition, reproductive success, and survival (Pan et al., 2010). Hence, we hypothesize that the ratio of body size of a larger over a smaller animal in the socialized environment reflects the extent to which the former exerts its *aggression* toward the latter. Accordingly, the body size difference of larger and smaller animals, divided by the body mass of the larger one, can be used as a surrogate for the strength of *altruism*. Based on the above-mentioned analysis, we derive mathematical descriptors to measure four types of intraspecific interactions, mutualism, antagonism, aggression, and altruism, by examining and comparing the body sizes of two interactive animals (Figure 1).

### Biological Justification of Interaction Measures

For a particular pair of animals in co-culture, we name the larger individual as L and the smaller individual as S. Let *w*_L_ and *w*_S_ denote the body size of L and S in co-culture, respectively. We argue that Figure 1's mathematical descriptors derived from *w*_L_ and *w*_S_ can measure the strengths of different interaction types that occur between the animals. To test these hypotheses, we analyze two real datasets, one from a cultural experiment of fish and the second from a published bacterial cultural study (Jiang et al., 2018). In each experiment, organisms were paired and two members in each pair were cultured both separately and jointly. Substantial evidence suggests that the organism often changes its phenotype in response to ecological interactions when it is shifted from an isolated environment to a socialized environment (Bohn and Amundsen, 2004, Fordyce, 2006, Lang and Benbow, 2013; Gamfeldt et al., 2013, Barraclough, 2015, Gracia-Lázaro et al., 2018). By quantifying the extent to which the phenotypic traits of the two individuals change from monoculture to co-culture, the strength of their ecological interaction can be measured and assessed.

We use *u*_L_ and *u*_S_ to denote the body size of individuals L and S in monoculture, respectively. Note that *u*_L_ is not necessarily greater than *u*_S_, although *w*_L_ is always greater than *w*_S_ by definition. If two individuals cooperate with each other, then the relative body size of each individual in co-culture over monoculture should not be less than 1.0 (Ghoul and Mitri, 2016). If one individual is aggressive on the other, i.e., the former grows at a cost of the latter, then the relative body size of the former over the latter would increase when the two individuals are relocated from their respective isolated environments to the common environment. Accordingly, if one individual is altruistic toward the other, i.e., the former sacrifices itself to benefit the latter, then the relative body size of the latter in co-culture over monoculture should be larger than the relative body size of the former in co-culture over monoculture. Based on these lines of reasoning, we use *M*_*u*_ = (*w*_L_/*u*_L_ + *w*_S_/*u*_S_)/2 to quantify the strength of mutualism between individuals L and S, *A*_*g*_ = (*w*_L_/*w*_S_)/(*u*_L_/*u*_S_) to quantify the strength of individual L's aggression toward individual S, and *A*_*l*_ = (*w*_S_/*u*_S_)/(*w*_L_/*u*_L_) to quantify the strength of individual L's altruism toward individual S.

#### Fish Experiment

We sampled five fish pairs from a population, in which the relative size of a smaller over larger one is 0.10, 0.38, 0.61, 0.80, and 1.00, with the larger one having a roughly similar size among pairs. Each pair was repeated four times. We reared each pair of fish in shared and isolated water buckets and measured their body mass 2 weeks after the fish was cultured. We calculated gains of body mass for each fish during culture.

**Figure 2:**
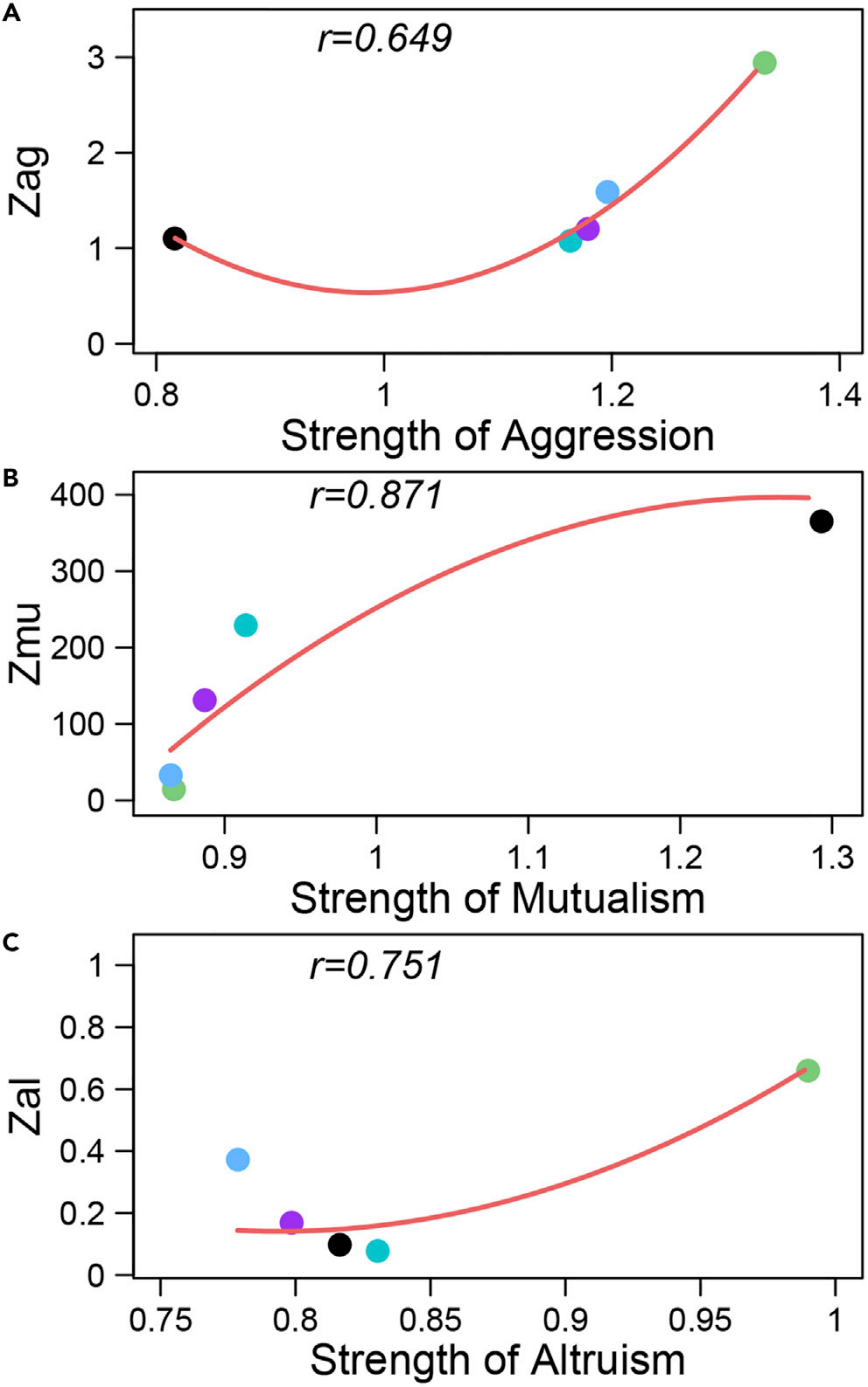
Biological Validation of Interaction Measures in a Fish Experiment Scatterplots of mathematical descriptors given in Figure 1 against the strength of ecological interactions across five different pairs of fish (dots) with relative body mass 0.10, 0.38, 0.61, 0.80, and 1.00. (A) Aggression descriptor (*z*_ag_) versus the strength of aggression. (B) Mutualism descriptor (*z*_mu_) versus the strength of mutualism. (C) Altruism descriptor (*z*_al_) versus the strength of altruism. The relationship between two variables is roughly fitted by a curve, with correlation coefficient (*r*) given within each plot.

Using the expressions given in Figure 1, we calculated and plotted parameters *z*_ag_, *z*_mu_, and *z*_al_ against *A*_*g*_, *M*_*u*_, and *A*_*l*_ for body mass gain across different fish pairs, respectively. We can test how well these three parameters can be used to measure the strengths of mutualism, parasitism, and altruism. It is interesting to find that *z*_ag_ is positively correlated with *A*_*g*_ (Figure 2A), thus suggesting that the former can approximately represent the strength of competition, especially the strength of aggression. We found that *z*_mu_ is positively correlated with *M*_*u*_ (Figure 2B), indicating that the former can well serve as a proxy to quantify the strength of mutualism. The positive correlation between *z*_al_ and *A*_*l*_ (Figure 2C) implies that the former is a good representation of the strength of altruism. From the above-mentioned analysis of fish data, it is suggested that the mathematical descriptors proposed can be used to measure different types of ecological interactions.

#### Microbial Experiment

Microbes have been widely used as a system to study ecological interactions (Damore and Gore, 2012). We further validated Figure 1's mathematical descriptors by re-analyzing a published bacterial data. Jiang et al. (2018) cultured two bacterial species, *Escherichia coli* and *Staphylococcus aureus*, in socialized and socially isolated conditions, respectively. They collected 45 diverse bacterial strains from each species. Each strain from one species was grown in monoculture and its interspecific pair with a randomly selected strain from the other species grown in co-culture. The abundance of each strain was measured once every 2 h during the first 24 h, followed by once every 4 h till 36 h, after the two types of culture were initiated.

**Figure 3:**
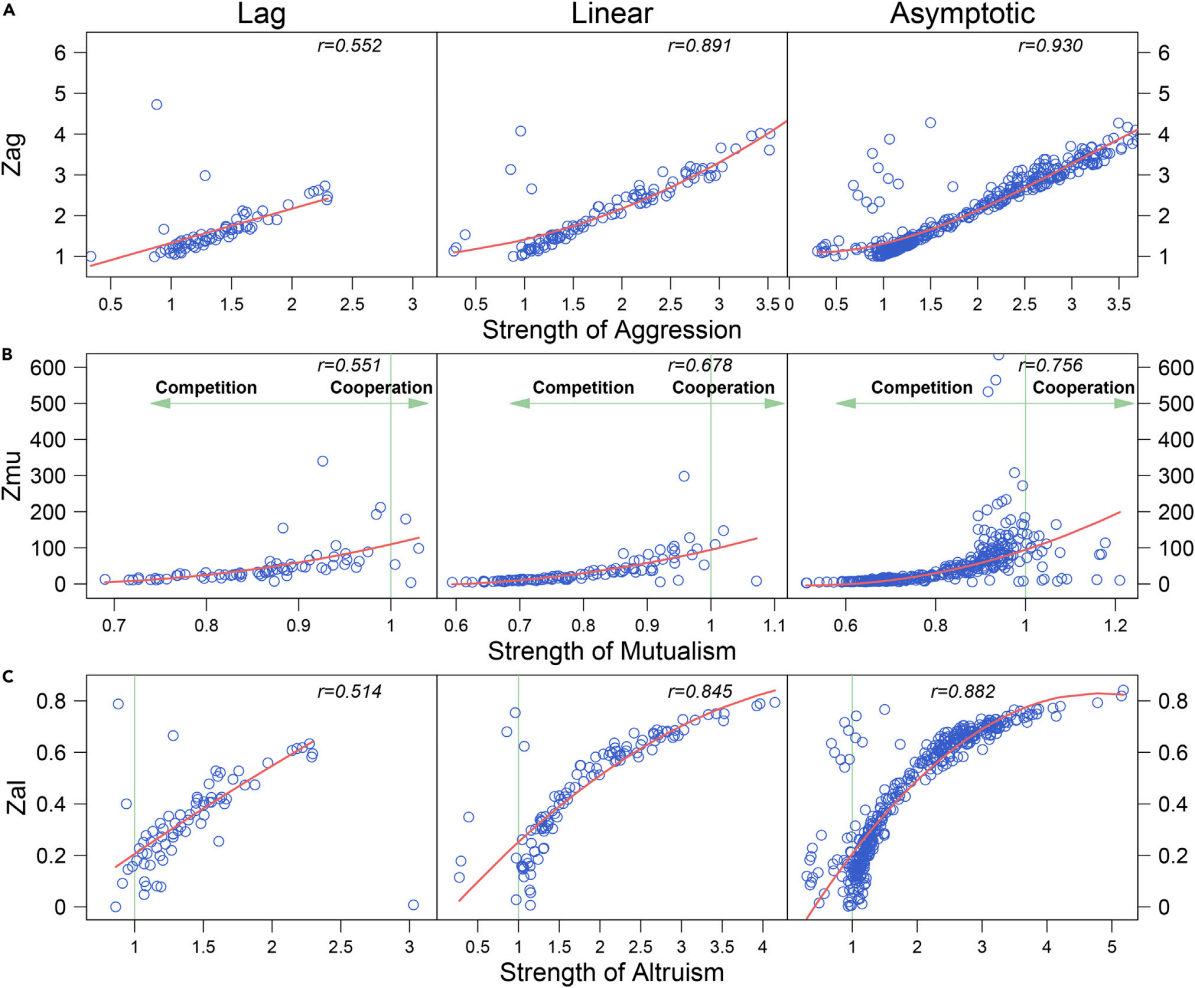
Biological Validation of Interaction Measures in a Bacterial Experiment Scatterplots of mathematical descriptors given in Figure 1 against the strength of ecological interactions across 45 interspecific pairs of strains from *E. coli* strains and *S. aureus* at three distinct phases of microbial growth (lag, linear, and asymptotic). (A) Aggression descriptor (*z*_ag_) versus the strength of aggression. (B) Mutualism descriptor (*z*_mu_) versus the strength of mutualism. The strength of mutualism is measured by the average of the ratio of abundance of each bacterial species in co-culture to monoculture. Thus, this ratio average quantifies the strength of cooperation if it is above 1 and the strength of competition if it is below 1. (C) Altruism descriptor (*z*_al_) versus the strength of altruism. Dots represent observations of different interspecific strain pairs at each time point. The relationship between two variables is roughly fitted by a curve, with correlation coefficient (*r*) given within each plot.

Organismic growth obeys a certain rule that can be described by a growth equation (West et al., 2001). We used an optimal growth equation to fit time-dependent abundance data of each strain and further partitioned its growth curve into lag, linear, and asymptotic phases (Zwietering et al., 1990). Using the mathematical expressions of Figure 1, we calculated parameters *z*_ag_, *z*_mu_, and *z*_al_ at each time points and plotted these parameters against *A*_*g*_, *M*_*u*_*,* and *A*_*l*_, respectively, estimated from co-culture and monoculture data across all strains. We found that *z*_ag_ is positively correlated with *A*_*g*_ (Figure 3A) (p < 0.01), showing the effectiveness of the former to measure the strength of aggression. These two variables display the strongest correlation at the asymptotic phase, followed by one at the linear and lag phases. This indicates that the ratio of a larger over smaller strain can better serve as a measure of the strength of aggression when the growth of strains tends to be stable. We found that *z*_mu_ is positively correlated with *M*_*u*_ (p < 0.01), especially at the asymptotic phase (Figure 3B; p < 0.001), suggesting that the former can be effectively used as the strength of cooperation. The *z*_mu_ values are much smaller in the competition zone (*M*_*u*_ < 1) than cooperation zone (*M*_*u*_ > 1). We found that *z*_al_ is positively correlated with *A*_*l*_ across strain pairs at three distinct phases, especially at linear and asymptotic phases (Figure 3C; p < 0.001), suggesting that the former can be effectively used as a proxy to measure the strength of altruism toward a larger individual from a smaller individual.

In summary, we formulate the mathematical descriptors of four typical ecological interactions, mutualism, antagonism, aggression, and altruism. We have carried out the fish experiment to validate the biological relevance of these descriptors, which was confirmed by a micribial experiment. A statistical model is implemented to map the genetic architecture of ecological interactions by treating these descriptors as phenotypes.

### Identification of Social QTL and Their Biological Relevance

The biological validation of the mathematical descriptors allows us to calculate and use four derived parameters, *z*_mu_, *z*_an_, *z*_ag_, and *z*_al_ (Figure 1), as measures of the strength of mutualism, antagonism, aggression, and altruism, respectively, between each pair of fish in our mapping population. We used these parameters to construct the networks of each interaction type. These ecological interaction networks were incorporated into the statistical framework of QTL mapping (see the Transparent Methods). Among 39,960 high-density SNPs (with an average marker distance of 0.75 cM), our model identified 158 QTL distributed over various chromosomes for body mass, including 80 acting through mutualism, 45 through antagonism, 98 through aggression, and 76 through altruism. Yet, no QTL for body mass can be detected by traditional approaches (Figure S1). We replicated the mapping experiment by generating two independent full-sib families G1 (n = 115, with 97,532 SNPs) and Z22 (n = 62, with 86,370 SNPs) from different common carp parents, from each of which a similar conclusion was reached; i.e., a number of QTL on different regions of the genome were detected by the ecological interaction-implemented model, whereas none was detected by traditional approaches (Figures S2 and S3).

We performed an extensive gene enrichment analysis for the significant SNPs by screening their up- and down-stream 10 kb regions on the sequenced genome of the common carp (Xu et al., 2014). Together, a large proportion of QTL detected from three mapping families were annotated to candidate genes: 86.2% for mutualism, 85.7% for antagonism, 85.9% for aggression, and 85.4% for altruism (Tables S1, S2, S3, and S4). We found that significant SNPs located in clusters are individually annotated to different genes (Figures S1–S3). All candidate genes have been previously reported in association with growth traits. For example, *pdlim4* (PDZ and LIM domain protein 4) of QTL carp227526 from family Z22 and *pdlim3* of QTL carp168806 from family G1 detected simultaneously by mutualistic, aggressive, and altruistic models are closely related to muscle growth and development (Hsieh et al., 2014). These three models also detected *gpc4* gene of carp028224 from family G1, *notch2* gene of QTL carp152585 from family Z22, and *gpr101* gene of QTL carp123609 from family G1. A family of *gpc* genes, e.g., *gpc1a, gpc3*, and *gpc4*, encoding glypicans, are expressed during the gastrulation stage of zebrafish, with their expression becoming more tissue specific and defined at the somitogenesis stages (Gupta and Brand, 2013). *notch2* has been widely reported to play a vital role in skeletal and muscle development (Zanotti and Canalis, 2013). *gpr101 gene* of QTL carp123609 from family G1 is involved in skeletal development (Beckers et al., 2015), and its other close GPR family members interact with *IGF*s and are crucial for muscle and body growth (Yang et al., 2014). Additionally, other genes identified uniquely by a certain model are also relevant in terms of biological functions; for instance, the genes *prss23* of carp170891 from family G1, *rarab* of carp055558 from family H1, *bmp1* of carp017510 from family H1, and *acer1* of carp117856 from family H1 were detected by the mutualistic, antagonistic, aggressive, and altruistic models, respectively. Molecular experiments in zebrafish showed that *prss23* was essential for endothelial-to-mesenchymal transition during valvulogenesis (Chen et al., 2013). Mice studies showed the involvement of *rarab* in fatty acid oxidation and energy homeostasis (Li et al., 2013). *bmp1* (bone morphogenetic protein 1) affects embryo development and osteogenesis (Muir et al., 2014) and is essential for human type 1 collagen fibrillogenesis (Valencia et al., 2014) *acer1* is important for mammalian skin homeostasis and the regulation of energy expenditure (Liakath-Ali et al., 2016).

To glean insight into the genetic mechanisms underlying the formation of body mass, we further performed GO and KEGG enrichment analyses for the QTL detected (Tables S5, S6, S7, and S8, Figures S4–S6). GO analysis identified significant enrichments of mutualism, aggression, and altruism QTL in “multicellular organism development (GO: 0007275)” and “fin development (GO: 003333),” both of which include two genes reported to affect zebrafish development, *notch2* (Zanotti and Canalis, 2013) and *hmcn1* (Feitosa et al., 2012). GO terms were enriched by the mutualistic model in “regulation of Notch signaling pathway (GO: 0008593),” which plays a vital role in bone and neurite development (Zanotti and Canalis, 2013). The antagonist model enriched “steroid hormone mediated signaling pathway (GO: 0043401)” (Li et al., 2013) and “B cell activation (GO: 0042113).” The enriched “B cell activation” suggests that stress-related genes, such as *prkcbb*, participate in fish-fish competition by regulating the *D*_*2*_*-like dopamine autoreceptor* (Luderman et al., 2015). “Somitogenesis (GO: 0001756),” uniquely identified by the aggressive model, is interestingly closely related to myogenesis and muscle growth (Gupta and Brand, 2013), which enhance the fish to develop a strong capacity for aggression. “Lipid metabolic process (GO: 0006629),” only detected by the altruistic model, is remarkably involved in energy expenditure (Liakath-Ali et al., 2016) and inhibits aggression, invoking altruism.

KEGG analysis found even more fascinating enriched pathways (Table S9). The mutualistic, aggressive, and altruistic models enriched four pathways closely associated with body weight: the “neuroactive ligand-receptor interaction,” “mTOR signaling pathway,” “progesterone-mediated oocyte maturation,” and “adrenergic signaling in cardiomyocytes.” For example, *gnai3* in the last pathway has been reported to regulate pig postnatal growth by engaging in miRNA-mRNA interactions (Ye et al., 2015). Mutualistic and altruistic models both identified the “Wnt signaling pathway,” which plays an important role in body axis patterning, cell proliferation, and cell migration and, therefore, embryonic development. These processes within the Wnt signaling pathway not only are necessary for bone and muscle formation but also control adult bone marrow, skin, and intestine tissue regeneration (Clevers et al., 2014), which is key to longevity and function.

### How QTL Act: Direct, Indirect, and Genome-Genome Epistatic Effects

**Figure 4:**
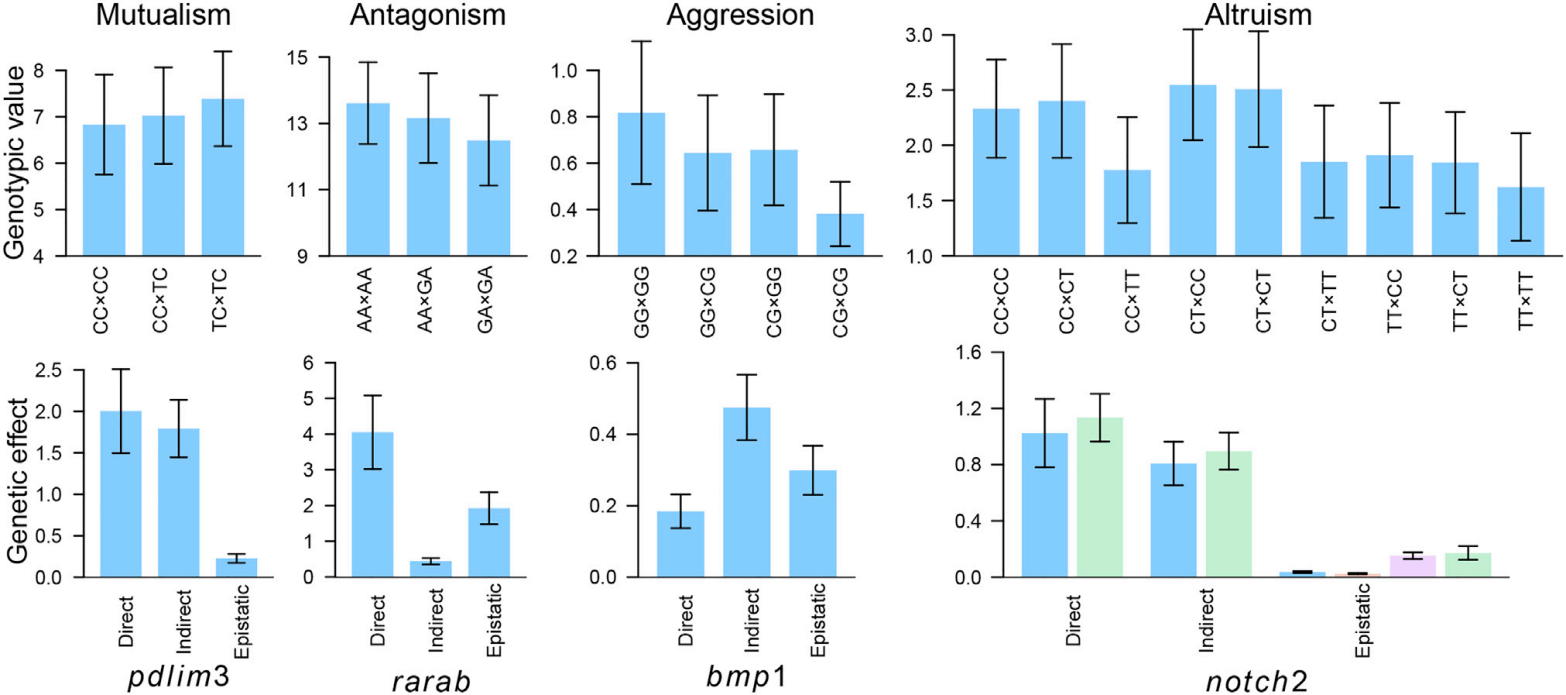
Quantitative Genetic Dissection of Genotype Combination Values For Fish Body Mass **Upper panel:** Genotypic values of combinations CC × CC, CC × TC, and TC × TC at *pdlim3* (testcross QTL) for the strength of mutualism; combinations AA × AA, AA × GA, and GA × GA at *thraa* (testcross QTL) for the strength of antagonism; combinations GG × GG, GG × CG, CG × GG, and CG × CG at *bmp1* (testcross QTL) for the strength of aggression; and combinations CC × CC, CC × CT, CC × TT, CT × CC, CT × CT, CT × TT, TT × CC, TT × CT, and TT × TT at *notch2* (intercross QTL) for the strength of altruism. **Lower panel:** Direct genetic effects that describe how the alleles of a fish in a pair affects its own body mass; indirect genetic effects that specify how each fish gene affects its conspecific's phenotypes; and genome-genome epistatic effects that quantify how the interactions between genes of two fish affect the phenotype of each fish. For the intercross QTL, both the direct and indirect effects include additive (blue) and dominant (green) effects and genome-genome epistatic effects include additive × additive, additive × dominant, dominant × additive, and dominant × dominant effects (in order from left to right). Standard errors for each value are given.

Our theory can partition the genotypic value of a social QTL into its different genetic components (see the Methods). *pdlim3* detected from family G1 is a testcross QTL for mutualism with two genotypes paired among the fish. The fish carrying the same genotype TC at this mutualism QTL are more cooperative with each other than with those carrying the alternative CC (Figure 4). At the *rarab* gene detected from family H1, stronger antagonism occurs between the fish of the same genotype AA than between those carrying different genotypes, and the fish with the same alternative genotype GA are the least antagonistic to each other. The fish carrying GG at *bmp1*, detected from family H1, repress those with the same genotype much more severely than with the alternative CG, whereas the fish of the same genotype CG are the least aggressive to each other. As an intercross QTL, the *notch2* detected from family H1, G1, and Z22, have three genotypes (CC, CT, and TT) forming nine genotype combinations among pairing fish. Genotype CT is more altruistic to the same genotype and genotype CC than to genotype TT, and genotype TT is the least altruistic to the same genotype among all combinations. Our model can separate the direct genetic effect of a QTL from one fish on its own body mass; the indirect genetic effect of a QTL from one fish on the body mass of its pairing partner; and the genome-genome epistatic effect of a QTL from two fish on the body mass of each fish. We found that mutualism *pdlim3* controls the body mass of fish not only through its direct effect but also through its indirect effect (Figure 4). The influence of genome-genome epistatic effect was evidently detected for antagonism *rarab*. Surprisingly, indirect and genome-genome epistatic effects are more pronounced than direct effect at aggression *bmp1*. As an intercross QTL, altruism *notch2* may exert its genetic impact by additive and dominant effects and their epistatic interactions. In fact, a remarkable indirect effect through both additive and dominant inheritance triggered by this QTL was found, although its genome-genome epistatic effects are not significant.

We further estimated the proportions of variance due to each of these effects to the total genetic variance at each QTL. Averaged over all QTL, indirect and genome-genome epistatic effects together explained approximately 50% of the total genetic variance for body mass, a phenomenon detected consistently in three mapping families (Table S10). These two portions of genetic components, largely neglected in previous quantitative and evolutionary genetic studies, may help geneticists chart a more complete genetic signature.

### Social Networks and QTL Networks

**Figure 5:**
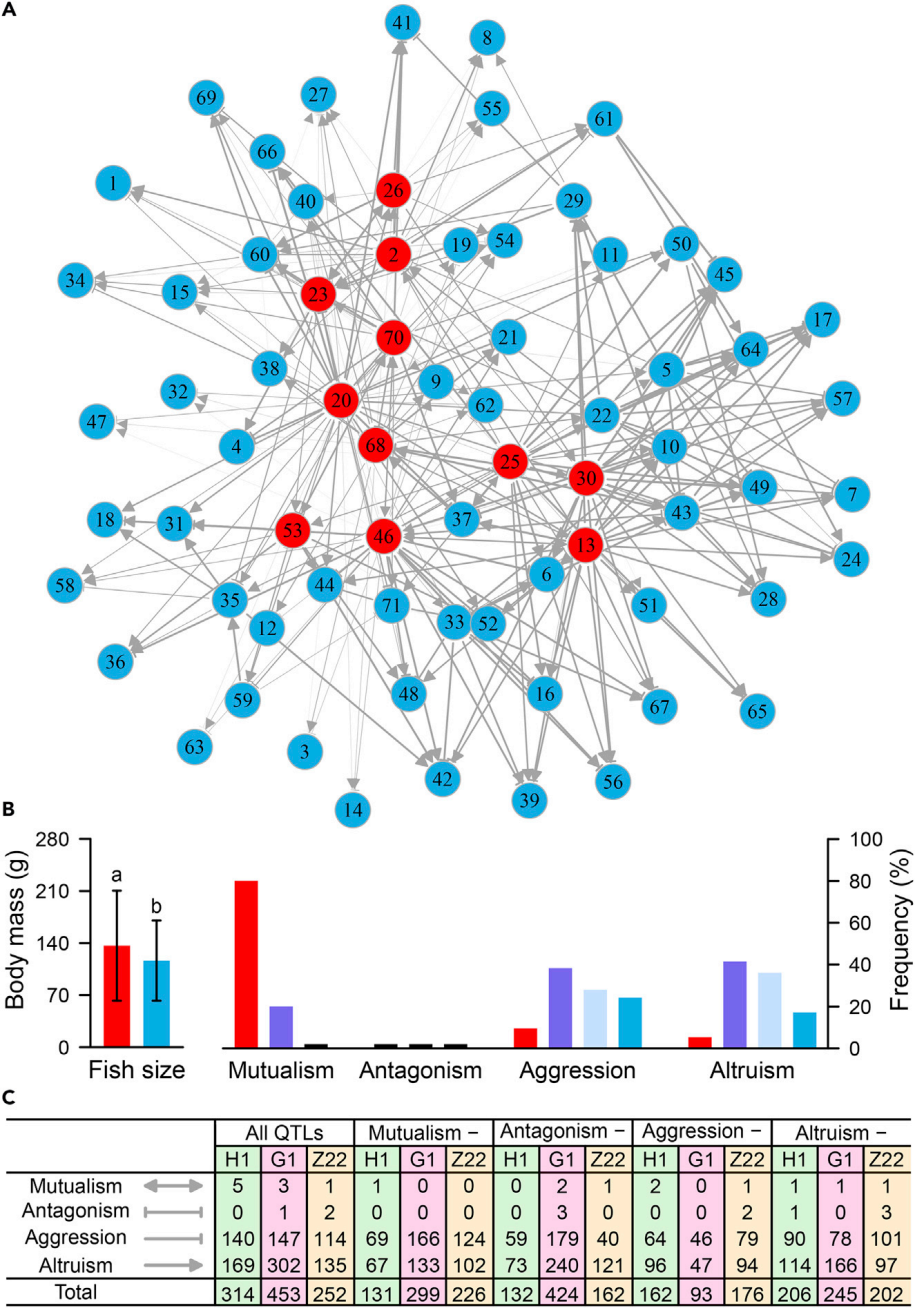
A Bidirectional, Signed, and Weighted Social Network of All Fish Driven by Various Types of QTL Constructed from Ordinary Differential Equations (A) Social network of family H1 constructed from all QTL with edges representing how one fish interacts with others through mutualism (doubly arrowed), antagonism (doubly T-shaped), aggression (singly T-shaped), or altruism (singly arrowed). Hubs of the network are highlighted in red. (B) The network is characterized by the difference in body mass between groups of hubs (red) and non-hubs (blue), the percentages of mutualistic and antagonistic edges among hubs (red), among hubs and non-hubs (purple), and among non-hubs, and the percentages of aggressive and altruistic edges from one fish to the second both from the hub group (red), from one fish from the hub group to the second from the non-hub group (purple), from one fish from the non-hub group to the second from the hub group (gray), and from one fish to the second both from the non-hub group (blue). (C) The numbers of mutualistic, antagonistic, aggressive, or altruistic edges with the social networks constructed from all QTL as well as from all QTL, except for, respectively, mutualism, antagonism, aggression, and altruism QTL. Comparisons of edge numbers are given not only for family H1, but also for the two family replicates G1 and Z22.

Using marginal genotypic values at each QTL, we modified an ordinary differential equation method (see the Methods) to infer a directed, signed, and weighted network of social interactions among the fish based on all QTL for mutualism, antagonism, aggression, and altruism. In the family H1 of 71 fish, this QTL-driven social network is composed of a total of 314 pillar connections from 2,485 possible links (Figure 5A), by which one fish connects and interacts with other fish selectively according to the game theory. For example, the network is dominated by 11 hub fish, which are larger than their marginal counterparts (p < 0.01) (Figure 5B). Of all mutualistic relationships, 80% occurs between the hub fish, 20% between the hub and marginal fish, and none between the marginal fish. The hub fish are less aggressive toward each other than toward the marginal conspecifics, although the marginal fish have some degree of aggression toward the hub fish and other marginal fish. The hub fish are also much less altruistic toward each other, compared with how much benefit they offer to the marginal ones. Similarly, the marginal fish are less altruistic toward each other than toward the hubs, although this difference is much more moderate compared with the difference detected in the hubs. All of these fish behaviors, which are consistent with the predictions from the game theory, suggest that animal's decision making in a socialized environment involves a strong genetic component.

In this study, we investigate how the underlying QTL govern behaviors of fish-fish interactions. We reconstructed four QTL-driven social fish networks by excluding either QTL for mutualism, or antagonism, or aggression, or altruism. The number of connections within each of these networks was, respectively, reduced sharply to 137, 132, 162, and 206 (Figure 5C), suggesting that a large number of QTL are essential for the maintenance of complex social networks. Specifically, when mutualism QTL were excluded, the number of mutualistic relationships was reduced to one, compared with five in the network constructed from all detected QTL. Similarly, aggressive relationships within the aggression QTL-excluded network and altruistic relationships within the altruism QTL-excluded network both become much less frequent (i.e., 64 and 114, respectively, compared with 140 and 169 within the network from all QTL). Similar findings have been confirmed in the other two families G1 and Z22 (Figure 5C). These results suggest that mutualism, aggression, and altruism QTL play an important role in forming and preserving, respectively, mutualistic, aggressive, and altruistic relationships in an interactive community. In other words, community structure, organization, and even function can be altered, modified, and engineered by activating, repressing, or removing the expression of specific social QTL.

**Figure 6:**
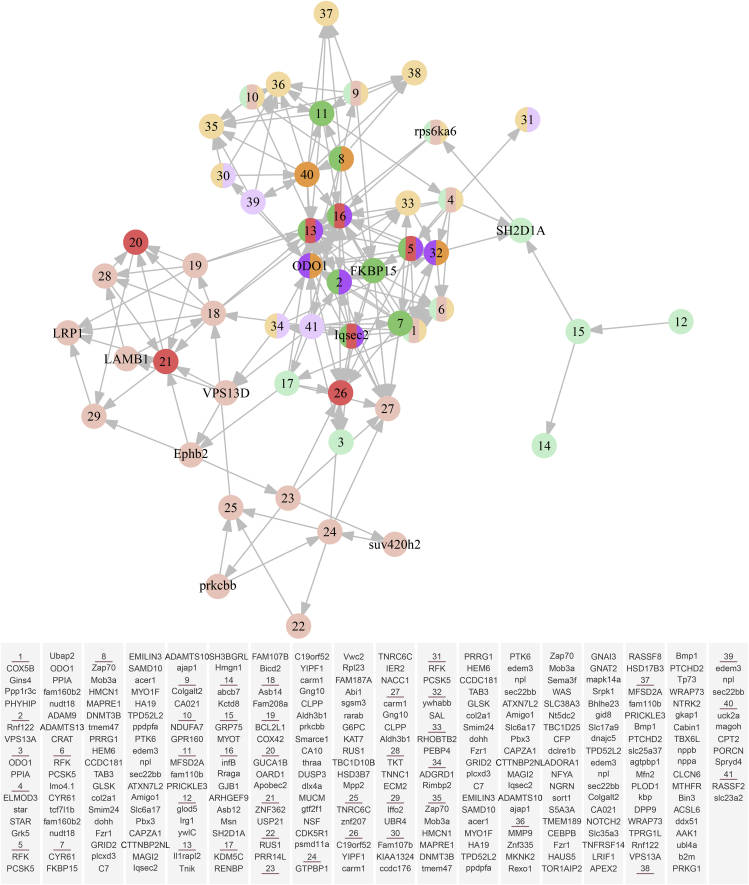
Dynamic Bayesian Genetic Network of All Detected QTL The entire network is dissolved into two distinct modules: one composed of mutualism QTL (green circle), aggression QTL (yellow circle), and altruism QTL (purple circle) and the other composed of antagonism QTL (red circle). The first module contains a proportion of QTL (mix-colored circle) that pleiotropically affect mutualistic, aggressive, and altruistic behaviors. In each module, hub QTL are highlighted in dark colors. Of all significant detected SNPs, 41 (each labeled by a number) were identified as uniquely segregating in the mapped population, which was used for QTL network construction. It is possible that different uniquely segregating SNPs may correspond to the same candidate gene if they are physically close enough on the common carp genome. Candidate genes adjacent to significant SNPs are listed below. Aggression *bmp1*, mutualism *hmcn1*, and antagonism *rarab* are annotated, respectively, by SNPs #37 and #38 and SNPs #35 and #24. The arrow denotes the direction by which one gene regulates the other.

To demonstrate how the detected QTL jointly affect the fish social network, we implemented ordinal Bayesian networks (see the Transparent Methods) to construct a directed acyclic graph (DAG) of QTL interactions for family H1 (Figure 6). We found that mutualism and antagonism QTL that determine two extreme patterns of social behaviors organize into distinct modules, connected via aggression and altruism QTL. A total of 10 QTL (*COX5B*, *STAR*, *ADAM9*, *LMO41*, *Iqsec2*, *Colgalt2*, *GPR160*, *Tnik*, *rps6ka6*, and *Msn*) pleiotropically affected the behavior of mutualism, aggression, and altruism. Other pleiotropic QTL included *VPS13A* for mutualism and aggression; *MYO1F* for mutualism and altruism; and *BBOF1*, *ODO1*, *RIFK*, *SAL*, and *AGRD1* for aggression and altruism. No QTL were detected to be shared for antagonism and the other types of interactions. Eleven QTL established a set of hub genes that modulate the structure and organization of the QTL network by activating or inhibiting other QTL. *bmp1* is socially an aggression QTL, but it is not genetically “aggressive” because its expression needs to be regulated by many other QTL. *hmcn1* affects fish mutualistic behavior, but its effect depends on the joint regulation of other QTL. Antagonism *rarab* is regulated by other genes, such as *suv420h2* and *prkcbb*, but it also modulates the expression of other genes. Overall, this QTL network helps to maintain the balance of social interactions by guiding the decision of individual fish to cooperate or compete with their conspecifics. Taken together, a detailed portrait of QTL DAG provides a mechanistic understanding of how QTL determine body mass in a fish population through their epistatic network. A similar phenomenon was also detected in families G1 and Z22, in which QTL form different but connected genetic modules according to their social behavior. These results, drawn consistently from three independent fish families, could provide evidence about the biological relevance of our theory.

### Monte Carlo Simulation

We examined the statistical behavior of our model through computer simulation. Our model estimates reasonably well the genetic effects of QTL, including direct, indirect, and genome-genome epistatic effects, and possesses good power for QTL detection (Table S11). This can be attributed to the increase of information from pairwise phenotypes under the design of our model. The same data were analyzed by a traditional model, which shows reduced power for QTL detection. The advantage of our model is more evident when the heritability and/or sample size are modest. The false-positive rates of our model are reasonably low (<0.08) even when the mapping population is modest (e.g., 70).

## Discussion

No organism can live in absolute isolation, rather the phenotype and fitness of an organism should be determined not only by its own intrinsic properties, but also by the strategies its conspecifics develop and use in response to the biological environment (Magnuson, 1962, Ribas et al., 2017, Schneider et al., 2017, Santostefano et al., 2017). However, measuring the strength of such ecological and social interactions from a mapping experiment is highly challenging. Based on animal behavioral ecology theory, we formulate the mathematical rule of thumb to quantitatively describe the strength of different interaction types that take place in a mapping population. The cultural experiments of fish and bacteria consistently support the biological relevance of our mathematical descriptors. We propose a mapping theory for complex traits by incorporating the mathematical descriptors of ecological interactions. We further arm our theory with a computational toolkit to map and identify QTL acting through direct genetic effects (by which an individual's QTL affects its own phenotype), indirect genetic effects (by which an individual's QTL influences the phenotype of its conspecifics), and trans-genome epistatic effects (by which the interaction of QTL derived from different individuals controls each of their phenotypes).

Our theory was used in three independent mapping experiments of fish, obtaining consistent results. We estimate the contributions of direct, indirect, and trans-genome epistatic genetic effects to quantitative genetic variation and find that the latter two effects can together account for approximately half of the total genetic variance in body mass. Many earlier studies have recognized the importance of indirect genetic effects (Schneider et al., 2017, Santostefano et al., 2017), but quantification of how they contribute to genetic variation has been lacking. Our mapping theory opens a gateway to capturing these overlooked sources of genetic variation, thereby portraying a more comprehensive genetic architecture of complex traits.

Apart from its increasing precision of trait mapping, our theory raises two key interdisciplinary questions for future research. First, quantitative genetic theory has been increasingly coupled with behavioral ecology to reveal the genetic mechanisms underlying social traits, such as aggression and response to social opponents (Dingemanse and Araya-Ajoy, 2015), and to uncover why selection maintains behavioral variation rather than eroding it (Santostefano et al., 2017). The major social interaction types of mutualism, antagonism, aggression, and altruism profoundly impact the structure and function of ecological communities in their unique ways. We found that these interactive processes have distinct genetic bases for fitness-related body size in the fish. To establish a complex social network, more QTL should be activated by playing a single or multifaceted role. By excluding mutualism, aggression, and altruism QTL, the fish become, respectively, less cooperative, aggressive, and altruistic in the population. This result has an immediate implication for the genetic study and possible manipulation in the real world of behavioral variation and evolution. By repressing or even eliminating the expression of aggression QTL through modern gene editing, such as CRISPR, researchers in ecology, breeding, or medicine can create and preserve more cooperative (e.g., for the gut microbiota) or more antagonistic (e.g., for intra-tumoral cells) communities beneficial to humans.

Second, indirect genetic effects arising from communal interactions are regarded as a source of additional genetic variation, whose impact on the social-traits evolutionary dynamics, by enhancing rapid selection responses or functioning as evolutionary constraint on phenotypes, has been well documented in many experimental studies (Wolf et al., 1998, Shuster et al., 2006, Wilson et al., 2011, Schneider et al., 2017, Santostefano et al., 2017). Not only are behavioral traits affected by indirect genetic effects, but also, as shown by our result, morphological traits, such as body mass, are influenced by an indirect genetic component. Our finding is innovative and insightful; for instance, we can infer through psychology that our human behavior responds indirectly to the presence of other surrounding humans' genes and their related-effects as these both affect our psyche and choices in food, which in turn then affect our body mass. The incorporation into evolutionary studies of these indirect genetic effects and trans-genome epistatic effects, expressed at specific QTL levels, can improve our insight into how social interactions between conspecifics impose a diverse array of selective pressures on various behaviors and how evolutionary stasis occurs for phenotypic traits involved in social interactions.

### Limitations of the Study

We propose a mapping theory for charting a more complete map of the genetic architecture of complex traits by incorporating the impact of ecological interactions on phenotypic variation. Although this theory has successfully identified the previously unknown genetic variation of body size in animals, it is unclear how it works to study and dissect other types of phenotypic traits, such as disease-related and physiological traits. Furthermore, our biological justification of interaction descriptors was based on cultural experiments of mobile animals and microbes, but we do not know whether this justification can be extended to immobile plants that communicate with each other differently from the way mobile organisms do. The unique feature of our theory is to take advantage of behavioral ecology to enhance the efficiency of trait mapping. The biological processes of how different organisms cooperate or compete for living resources in populations, communities, or ecosystems are also governed by evolutionary principles, developmental biology, habitat ecology, and network science. The seamless integration of all these disciplines into our mapping theory will certainly facilitate its widespread use to construct mechanistic links from genotype to phenotype.

## Methods

All methods can be found in the accompanying Transparent Methods supplemental file.

## Data and Code Availability

All data and computer code are given in GitHub https://github.com/LiboJiang/Fish or can be directly requested from the corresponding author.

## Supplemental Information

Document S1. Transparent Methods, Figures S1–S7, and Tables S10–S14

Table S1. Gene Annotation of Significant SNPs for Body Mass Detected by the Mutualistic Model, Related to Figures 4–6

Table S2. Gene Annotation of Significant SNPs for Body Mass Detected by an Antagonistic Model, Related to Figures 4–6

Table S3. Gene Annotation of Significant SNPs for Body Mass Detected by an Aggressive Model, Related to Figures 4–6

Table S4. Gene Annotation of Significant SNPs for Body Mass Detected by an Altruistic Model, Related to Figures 4–6

Table S5. Gene Ontology Enrichment of Body Mass-Related Genes Detected by a Mutualistic Model, Related to Figures 4–6

Table S6. Gene Ontology Enrichment of Body Mass-Related Genes Detected by an Antagonistic Model, Related to Figures 4–6

Table S7. Gene Ontology Enrichment of Body Mass-Related Genes Detected by an Aggressive Model, Related to Figures 4–6

Table S8. Gene Ontology enrichment of body mass-related genes detected by an altruistic model, related to Figures 4–6

Table S9. KEGG Enrichment of Body Mass-Related Genes Detected by Different Models, Related to Figures 4–6
