# Supplementary material for: A Drive to Driven Model of Mapping Intraspecific Interaction Networks
Source: iScience. 2019 Nov 6;22:109–22. doi: 10.1016/j.isci.2019.11.002 (PMC6883333; doi:10.1016/j.isci.2019.11.002)
Supplement: Document S1. Transparent Methods, Figures S1–S7, and Tables S10–S14 [file mmc1.pdf]

ISCI, Volume *22*

Supplemental Information

A Drive to Driven Model of Mapping

Intraspecific Interaction Networks

Libo Jiang, Jian Xu, Mengmeng Sang, Yan Zhang, Meixia Ye, Hanyuan Zhang, Biyin Wu, Youxiu Zhu, Peng Xu, Ruyu Tai, Zixia Zhao, Yanliang Jiang, Chuanju Dong, Lidan Sun, Christopher H. Griffin, Claudia Gragnoli, and Rongling Wu

Supplementary Figures

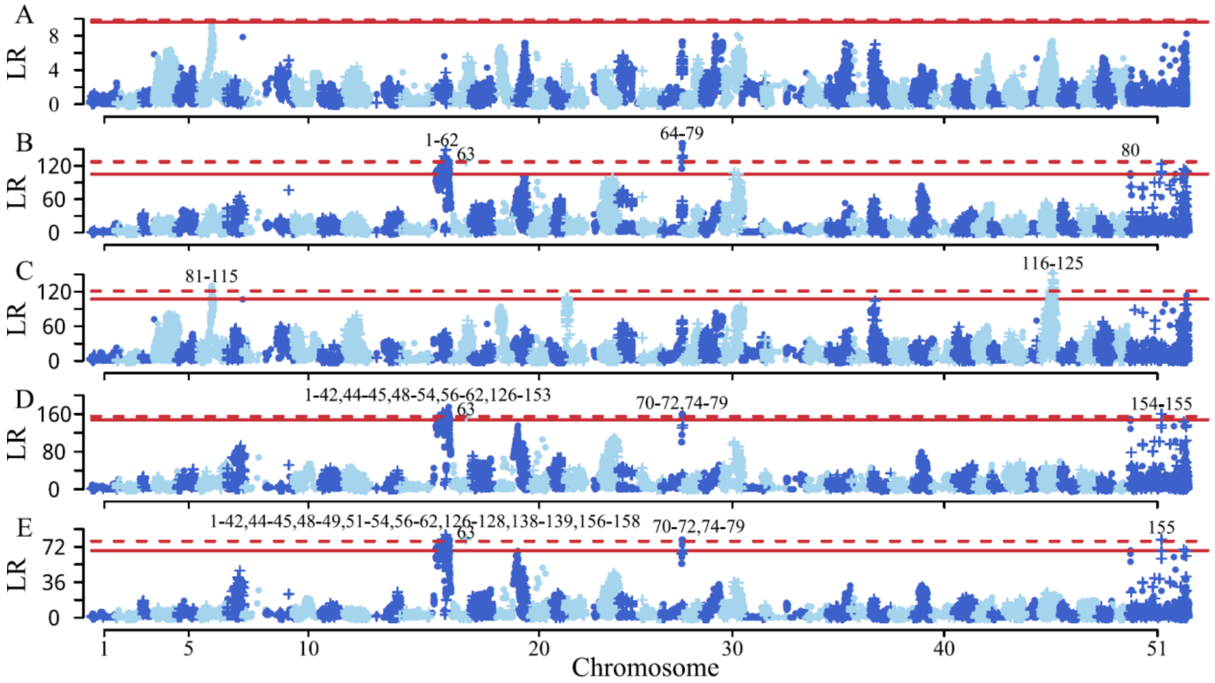

	SNP	Gene		SNP	Gene		SNP	Gene		SNP	Gene		SNP	Gene		SNP	Gene		SNP	Gene
1	023349	MAPRE1	20	066847	GLSK	43	085983	RENBP	64	190216	abcb7	90	146379	thraa	111	084168	G6PC	136	107548	PEBP4
1	023349	DNMT3B	20	066847	col2a1	43	085983	FAM107B	65	014339	glod5	90	146379	DUSP3	111	084168	KAT7	137	152452	Bin3
2	023400	Sema3f	21	238568	ajap1	43	085983	Bicd2	65	014339	Irg1	90	146379	dlx4a	111	084168	RUS1	137	152452	ACSL6
2	023400	WAS	22	229654	TNFRSF14	44	103111	plcxd3	65	014339	ywlC	91	044586	TNRC6C	112	084338	RUS1	138	101022	ODO1
2	023400	SLC38A3	22	229654	LRIF1	44	103111	C7	66	229327	Kctd8	92	044587	TNRC6C	112	084338	PRR14L	139	006127	RFK
3	023418	Nt5dc2	22	229654	APEX2	45	087990	lmo4.1	67	013816	GRP75	93	044589	TNRC6C	113	084374	TBC1D10B	139	006127	PCSK5
3	023418	TBC1D25	22	229654	RASSF8	45	087990	CYR61	68	013814	GRP75	94	044591	TNRC6C	114	084384	HSD3B7	140	094524	gkap1
3	023418	CFP	23	229571	Iqsec2	46	087997	CYR61	69	013849	GRP75	95	044595	TNRC6C	115	160699	suv420h2	141	081988	NTRK2
4	126966	mapk14a	24	229561	MAGI2	46	087997	FKBP15	69	013849	MYOT	96	044599	TNRC6C	116	063495	LRP1	142	081992	agtpbp1
4	126966	Srpk1	25	103673	EMILIN3	47	088008	FKBP15	70	241838	Rraga	96	044599	znf207	117	217807	Ephb2	143	160271	ddx51
5	030825	dclre1b	25	103673	SAMD10	48	088100	tcf7l1b	70	241838	GJB1	97	044601	TNRC6C	118	011814	VPS13D	144	160274	ddx51
5	030825	ADORA1	26	133836	Bhlhe23	49	188586	COX5B	71	211157	infB	97	044601	znf207	119	110562	Iffo2	144	160274	AAK1
6	050747	TAB3	26	133836	gid8	49	188586	Gins4	72	243252	rps6ka6	98	044557	C19orf52	119	110562	UBR4	145	160276	ddx51
6	050747	tmem47	26	133836	Slc17a9	49	188586	Ppp1r3c	73	243233	SH2D1A	98	044557	YIPF1	120	067893	ZNF362	145	160276	AAK1
7	050738	tmem47	27	133842	Slc17a9	49	188586	PHYHIP	74	243228	SH2D1A	98	044557	carm1	120	067893	USP21	146	064960	Tp73
7	050738	PRRG1	27	133842	dnajc5	50	188600	Rnf122	74	243228	ARHGEF9	99	044561	C19orf52	121	079040	LAMB1	146	064960	WRAP73
7	050738	HEM6	27	133842	TPD52L2	50	188600	VPS13A	75	243221	ARHGEF9	99	044561	YIPF1	122	206556	TKT	147	170472	WRAP73
7	050738	CCDC181	28	133915	TPD52L2	51	200245	ELMOD3	76	243217	ARHGEF9	99	044561	carm1	122	206556	TNNC1	147	170472	TPRG1L
8	134195	Colgalt2	28	133915	ppdpfa	51	200245	star	76	243217	Asb12	100	044562	C19orf52	122	206556	ECM2	148	094144	PLOD1
8	134195	CA021	28	133915	PTK6	51	200245	Grk5	76	243217	Msn	100	044562	YIPF1	123	218909	Asb14	149	084967	Mfn2
9	134194	Colgalt2	29	012927	MMP9	52	101059	fam160b2	77	176389	SH3BGRL	100	044562	carm1	123	218909	Fam208a	150	084964	Mfn2
9	134194	CA021	29	012927	Znf335	52	101059	nudt18	77	176389	Hmgn1	101	044576	carm1	124	080883	GUCA1B	151	132829	nppb
10	134185	edem3	30	199302	Slc6a17	52	101059	nudt18	78	167407	Tnik	101	044576	Gng10	124	080883	OARD1	151	132829	nppa
10	134185	npl	31	037443	NFYA	53	101054	fam160b2	78	167407	Il1rapl2	101	044576	CLPP	124	080883	Apobec2	151	132829	CLCN6
10	134185	sec22bb	31	037443	NGRN	53	101054	nudt18	79	096899	Il1rapl2	101	044576	Aldh3b1	125	040975	BCL2L1	152	137777	MTHFR
11	134184	edem3	31	037443	sort1	53	101054	nudt18	80	172289	MFSD2A	102	044578	carm1	125	040975	COX42	153	132788	kbp
11	134184	npl	32	199145	ATXN7L2	53	101054	ADAM9	80	172289	fam110b	102	044578	Gng10	126	126958	Fam107b	153	132788	DPP9
11	134184	sec22bb	32	199145	Amigo1	54	101018	ODO1	80	172289	PRICKLE3	102	044578	CLPP	126	126958	KIAA1324	154	005538	MFSD2A
12	164663	NOTCH2	33	100657	GNAI3	54	101018	PPIA	81	132961	prkcbb	102	044578	Aldh3b1	126	126958	ccdc176	154	005538	fam110b
12	164663	Slc35a3	33	100657	GNAT2	55	101017	ODO1	82	132960	prkcbb	103	166452	MUCM	127	134101	Rimbp2	154	005538	PRICKLE3
13	117856	acer1	33	100657	GNAT2	55	101017	PPIA	83	132959	prkcbb	103	166452	gtf2f1	128	132232	ADGRD1	155	060805	ywhabb
13	117856	MYO1F	34	244199	HSD17B3	56	006120	RFK	84	132955	prkcbb	104	085722	CACNA1A	129	017510	Bmp1	155	060805	SAL
14	229992	ADAMTS10	35	100674	GRID2	56	006120	PCSK5	84	132955	Smarce1	105	163753	IER2	129	017510	PTCHD2	156	134183	edem3
15	018681	Zap70	36	225167	CAPZA1	57	006124	RFK	84	132955	CA10	105	163753	NACC1	130	017511	Bmp1	156	134183	npl
15	018681	Mob3a	36	225167	CTTNBP2NL	57	006124	PCSK5	85	190923	CDK5R1	106	163750	IER2	130	017511	PTCHD2	156	134183	sec22bb
16	018682	Zap70	37	225164	CAPZA1	58	006126	RFK	85	190923	psmd11a	106	163750	NACC1	131	093309	PTCHD2	157	066853	uck2a
16	018682	Mob3a	37	225164	CTTNBP2NL	58	006126	PCSK5	86	190917	CDK5R1	107	009718	sgsm3	132	199061	ubl4a	157	066853	magoh
17	018642	MKNK2	38	037549	S5A3A	59	006130	PCSK5	86	190917	psmd11a	107	009718	GTPBP1	132	199061	b2m	157	066853	CPT2
17	018642	Rexo1	38	037549	TMEM189	60	067130	Ubap2	87	190912	NSF	108	009708	GTPBP1	132	199061	PRKG1	157	066853	PORCN
18	066881	Smim24	38	037549	CEBPB	61	132578	ADAMTS13	88	146382	thraa	108	009708	Vwc2	133	188601	Rnf122	157	066853	Spryd4
18	066881	dohh	39	022408	HMCN1	61	132578	CRAT	88	146382	DUSP3	108	009708	Rpl23	133	188601	VPS13A	158	247828	RASSF2
18	066881	Fzr1	40	217376	Pbx3	62	069836	NDUFA7	88	146382	dlx4a	108	009708	FAM187A	134	116911	Cabin1	158	247828	slc23a2
19	066875	Fzr1	41	132736	HA19	62	069836	GPR160	89	146381	thraa	108	009708	Abi1	134	116911	TBX6L			
19	066875	HAUS5	42	086001	Iqsec2	63	006121	RFK	89	146381	DUSP3	109	177182	Mpp2	135	035665	slc25a37			
19	066875	TOR1AIP2	43	085983	KDM5C	63	006121	PCSK5	89	146381	dlx4a	110	055558	rarab	136	107548	RHOBTB2			

Figure S1. Manhattan plot of log-likelihood ratios (LR) for testing significant SNPs, including testcross markers (+) and intercross markers (•), throughout the common carp genome in family H1 by the traditional mapping model (**A**) and our mapping model (**B** – **E**), related to Figures 4–6. Whereas the former did not discoevr any significant QTL, the latter has identified a number of significant loci for mutualism (**B**), antagonism (**C**), aggression (**D**), and altruism (**E**). The genome-wide critical threshold at the 5% significance level, indicated by solid lines for testcross markers and broke lines for intercross markers, was determined by 10,000 permutation tests. Through GO analysis, significant QTL for different types of social interactions, labelled by 1 – 158, are annotated by candidate genes with names given in the lower panel. Among a total of 158 QTL detected, 80 are for mutualism, 45 for antagonism, 98 for aggression and 76 for altruism, with a portion of QTL that pleiotropically affect more than one interaction type.

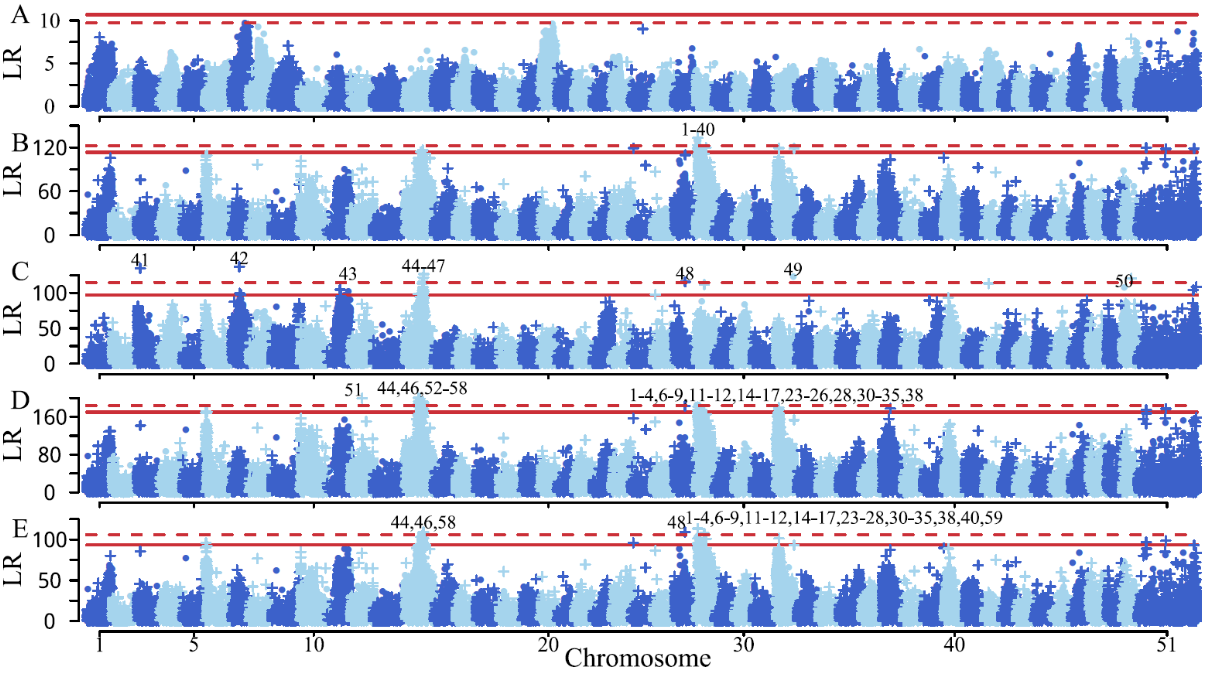

	SNP	Gene		SNP	Gene		SNP	Gene		SNP	Gene		SNP	Gene		SNP	Gene		SNP	Gene
1	168815	CD047	9	170939	FOXN3	17	181029	Phf6	25	187399	MAP7	34	004863	ATP11C	40	198966	spdl1	51	033423	cp045
2	168806	PDLIM3	9	170939	ATL3	18	170052	Phf6	26	187405	MAP7	35	164051	P2RY4	40	198966	HNRNPA0	52	151805	NFAC3
2	168806	Sorbs2	9	170939	Cfl2	18	170052	HPRT1	27	008690	OPSP	35	164051	SHP2B	41	130075	PRKAG2	53	041393	TPPP3
3	168843	Sorbs2	10	170891	Cfl2	18	170052	FAM122A	27	008690	CD40LG	36	164026	NKRF	41	130075	Bcl2	53	041393	Dpep2
4	128618	Fam149a	10	170891	Prss23	19	170041	FAM122A	27	008690	ARHGEF6	36	164026	Ube2a	42	242720	K1644	54	179202	TNNT3
4	128618	Cyp4v2	11	170755	FOXO1	19	170041	Mospd1	28	123605	RBMX	37	223337	LNX2	43	081258	NBEAL1	54	179202	Prr33
5	035590	ASAH1	12	222839	FOXO1	19	170041	ints6	28	123605	TM9SF2	37	223337	CHIC1	43	081258	Stradb	54	179202	Lsp1
5	035590	FGL1	12	222839	SESN3	20	169918	FAM122A	28	123605	GPR101	37	223337	Cdx4	44	109517	B4GALNT4	55	127809	Slc7a10
5	035590	mtus1a	13	170234	Gpc4	20	169918	Mospd1	29	123609	TM9SF2	38	020386	Dlg3	45	007683	BRSK2	56	128833	CadN
6	171940	HE71a	14	028224	Gpc4	20	169918	ints6	29	123609	GPR101	38	020386	GDPD5	46	007093	ADAMTSL5	57	129279	Sin3a
6	171940	HE71a	14	028224	Gpc3	21	099486	ints6	30	004841	MCF2L	39	172990	GDPD5	47	082193	PPIP5K1	58	129371	Kcnq1
6	171940	Pfdn6	15	028220	Gpc3	22	099490	ints6	31	004780	MCF2L	39	172990	pdzd11	47	082193	FMT	58	129371	CDKN1C
7	171892	HE71a	16	181027	Gpc3	22	099490	mmgt1	31	004780	ATP11C	39	172990	STARD10	48	228905	sept8b	58	129371	TRPM5
7	171892	HE71a	16	181027	CC160	22	099490	SLC9A6	32	004846	MCF2L	39	172990	RAB6A	48	228905	SFXN1	59	069271	ZDHHC5
7	171892	Pfdn6	16	181027	CAB39	23	187391	Fhl1	32	004846	ATP11C	40	198966	csnk1a1	49	201440	skap2	59	069271	sept8b
7	171892	RM11	17	181029	CC160	23	187391	MAP7	33	004854	MCF2L	40	198966	SPINK1	50	246587	Brsk2			
8	171706	npas4	17	181029	CAB39	24	187398	MAP7	33	004854	ATP11C	40	198966	SIL1	51	033423	PIK3R6			

Figure S2. Manhattan plot of log-likelihood ratios (LR) for testing significant SNPs, including testcross markers (+) and intercross markers (•) throughout the common carp genome in family G1 by the traditional mapping model (**A**) and our mapping model (**B** – **E**), related to Figures 4–6. Whereas the former did not discoevr any significant QTL, the latter has identified a number of significant loci for mutualism (**B**), antagonism (**C**), aggression (**D**), and altruism (**E**). The genome-wide critical threshold at the 5% significance level, indicated by solid lines for testcross markers and broke lines for intercross markers, was determined by 10,000 permutation tests. Through GO analysis, significant QTL for different types of social interactions, labelled by 1 – 59, were annoated by candidate genes with names given in the lower panel. Among a total of 59 QTL detected, 40 are for mutualism, 10 for antagonism, 36 for aggression and 33 for altruism, with a portion of QTL that pleiotropically affect more than one interaction type.

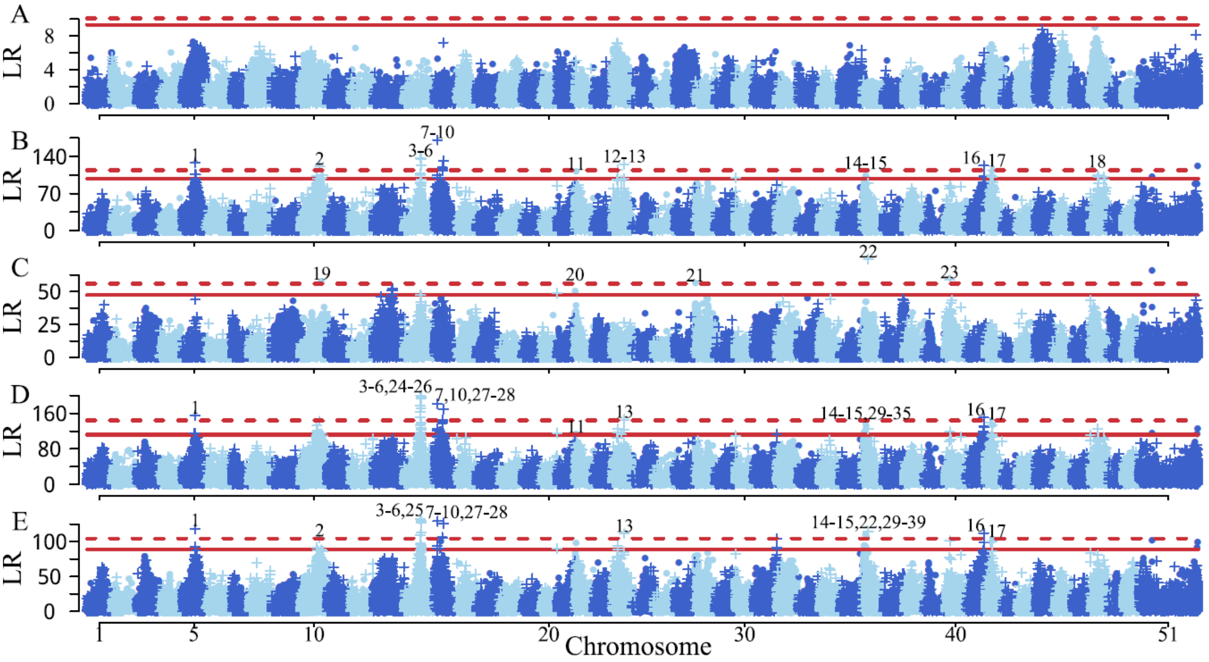

	SNP	Gene		SNP	Gene		SNP	Gene		SNP	Gene		SNP	Gene		SNP	Gene		SNP	Gene
1	096344	Tnrc6a	8	152585	Slc35a3	15	026099	Shank3	21	118724	MTR1A	27	017630	MIER3	32	093914	phrA	37	166754	PTPRQ
2	108447	ACAC	8	152585	Fam78b	16	227526	PDLIM4	21	118724	SLC36A1	28	117857	MLLT1	32	093914	GCSH	38	047707	MOB2
3	209675	THRB	9	066501	NFIC	17	116595	Rpl17	22	236365	WFDC1	28	117857	acer1	32	093914	OTOGL	38	047707	Ppp6r2
3	209675	ZNF408	10	120428	mad2l2	17	116595	Nkx6-1	22	236365	CIRH1A	29	032971	ZBBX	33	085416	Shank3	39	085427	Shank3
4	236914	celf1	11	232795	PHACTR3	18	220161	Map7d2	22	236365	VPS9D1	30	093923	ZFHX3	33	085416	Shank3			
5	201126	RNF182	12	166997	llgl2	18	220161	CXorf23	23	242462	RHAG	31	093913	phrA	34	085422	Shank3			
6	127830	Abcc12	13	193943	Arhgap17	18	220161	liph	24	179229	Otog	31	093913	phrA	34	085422	Shank3			
7	026598	trim33	13	193943	grid2ip	19	210271	MAT2A	24	179229	TNNT3	31	093913	GCSH	35	154356	Shank3			
7	026598	PQLC2	14	205945	Cd9	20	163687	col2a1	25	020799	AMBRA1	31	093913	OTOGL	36	243035	ARHGEF17			
8	152585	NOTCH2	14	205945	VWF	20	163687	MYG1	26	027378	CREB3L1	32	093914	phrA	36	243035	P2RY3			

Figure S3. Manhattan plot of log-likelihood ratios (LR) for testing significant SNPs, including testcross markers (+) and intercross markers (•) throughout the common carp genome in family Z22 by the traditional mapping model (**A**) and our mapping model (**B** – **E**), related to Figures 4–6. Whereas the former did not discover any significant QTL, the latter has identified a number of significant loci for mutualism (**B**), antagonism (**C**), aggression (**D**), and altruism (**E**). The genome-wide critical threshold at the 5% significance level, indicated by solid lines for testcross markers and broke lines for intercross markers, was determined by 10,000 pertumtation tests. Through GO analysis, significant QTL for different types of social interactions, labelled by 1 – 39, were annoated to candidate genes with names given in the lower panel. Among a total of 39 QTL detected, 18 are for mutualism, 5 for antagonism, 25 for aggression and 31 for altruism, with a portion of QTL that pleiotropically affect more than one interaction type.

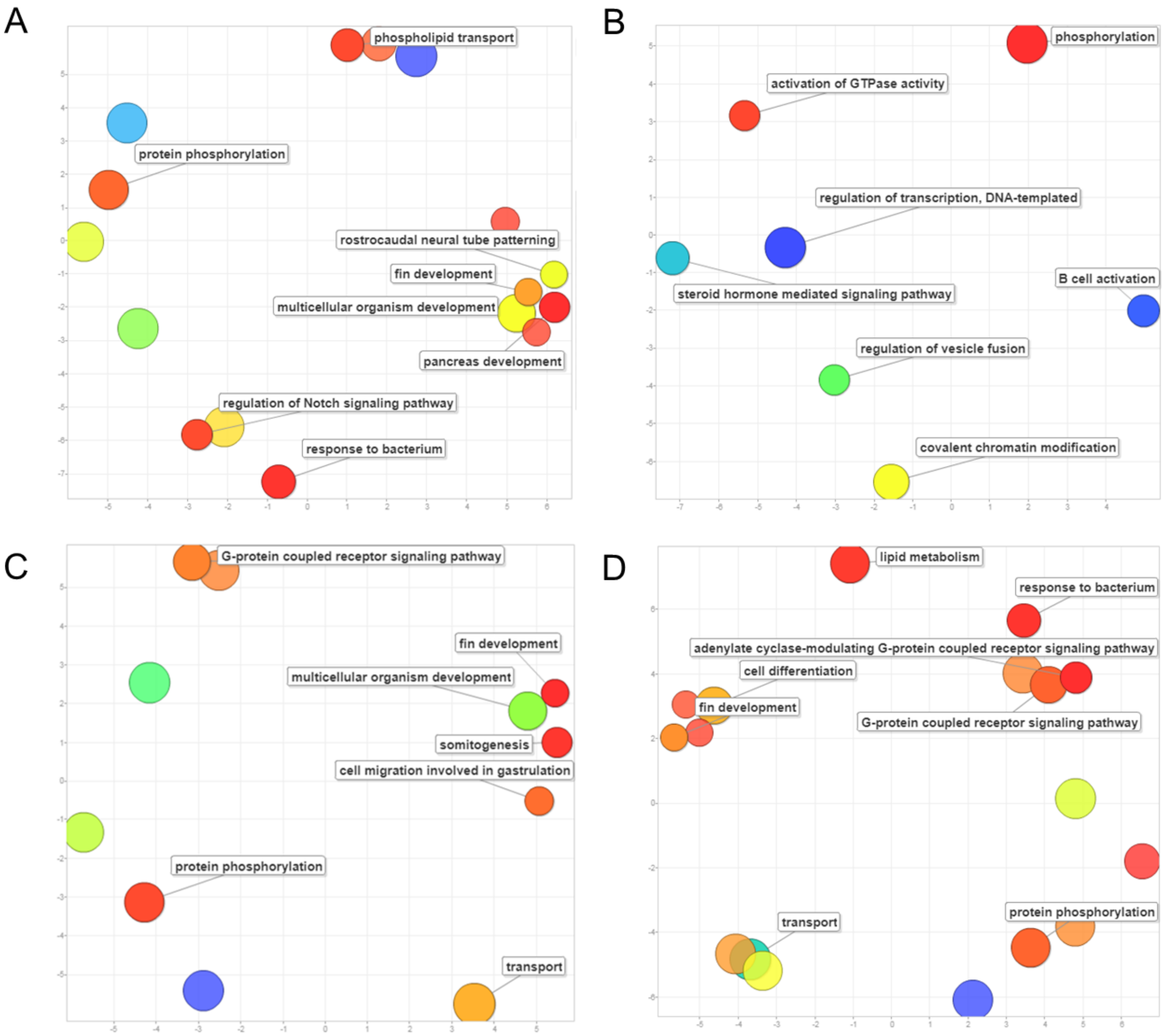

Figure S4. Biological processes of GO terms enriched in mutualism QTL (A), antagonism QTL (B), aggression QTL (C), and altruism QTL (D) for body mass, related to Figures 4–6.
Colors of the bubbles represent the significance level of GO terms. The size of bubbles represents the gene numbers involved in each GO term. X and Y dimensions represent the semantic spaces among different GO terms.

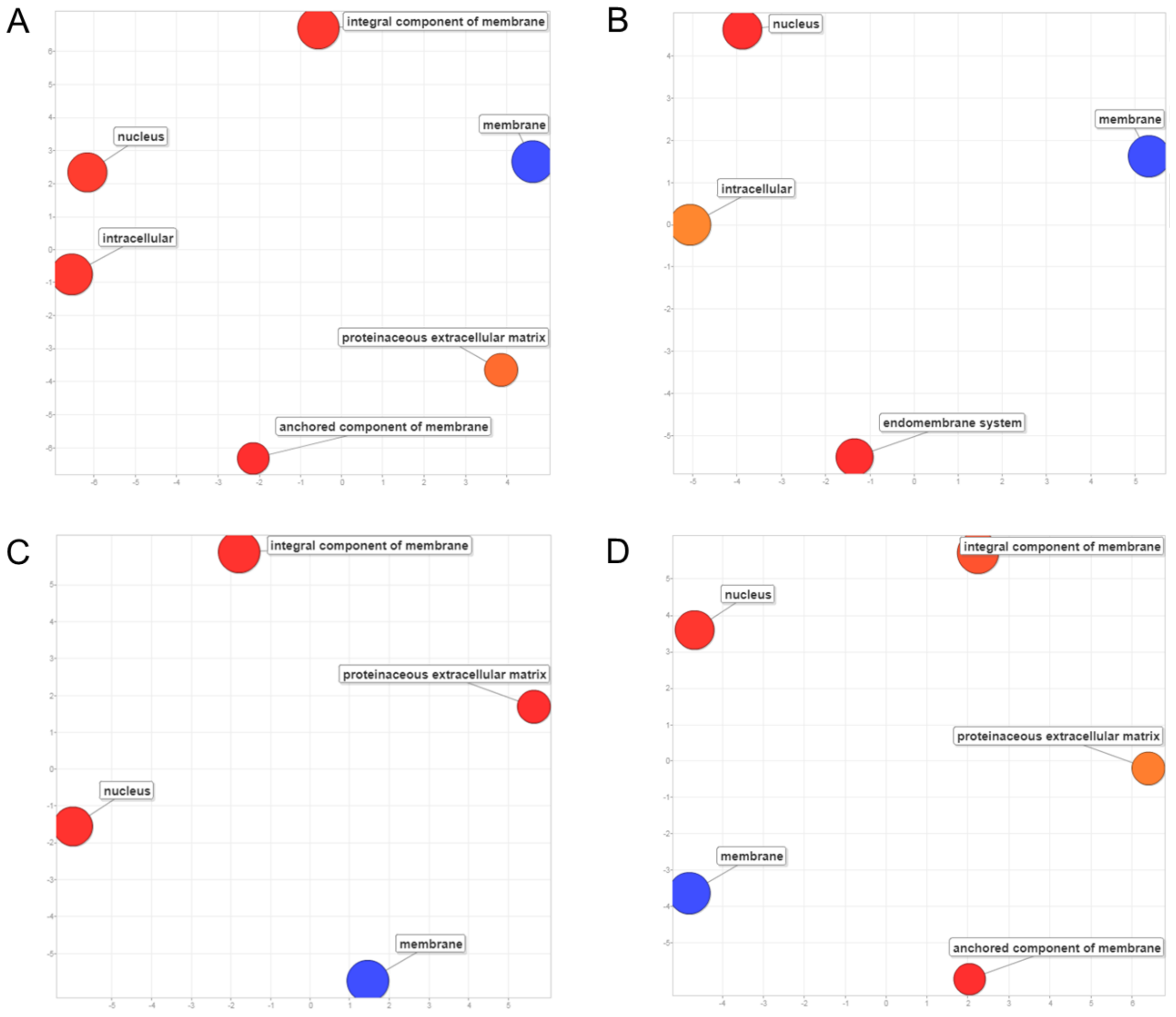

Figure S5. Cellular components of GO terms enriched in mutualism QTL (A), antagonism QTL (B), aggression QTL (C), and altruism QTL (D) for body mass, related to Figures 4–6.
Colors of the bubbles represent the significance level of GO terms. The size of bubbles represents the gene numbers involved in each GO term. X and Y dimensions represent the semantic spaces among different GO terms.

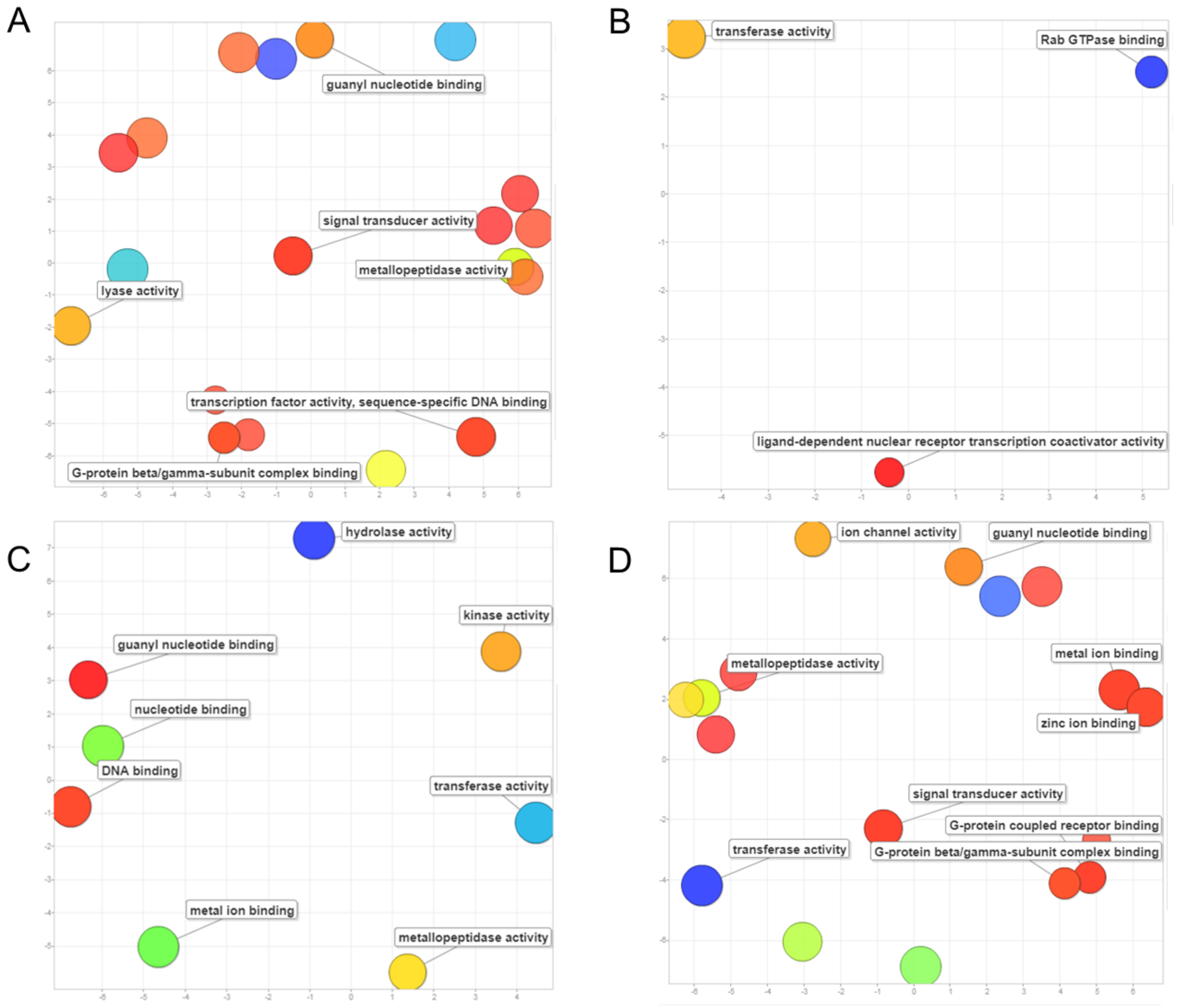

Figure S6. Molecular functions of GO terms enriched in mutualism QTL (A), antagonism QTL (B), aggression QTL (C), and altruism QTL (D) for body mass, related to Figures 4–6.

Colors of the bubbles represent the significance level of GO terms. The size of bubbles represents the gene numbers involved in each GO term. X and Y dimensions represent the semantic spaces among different GO terms.

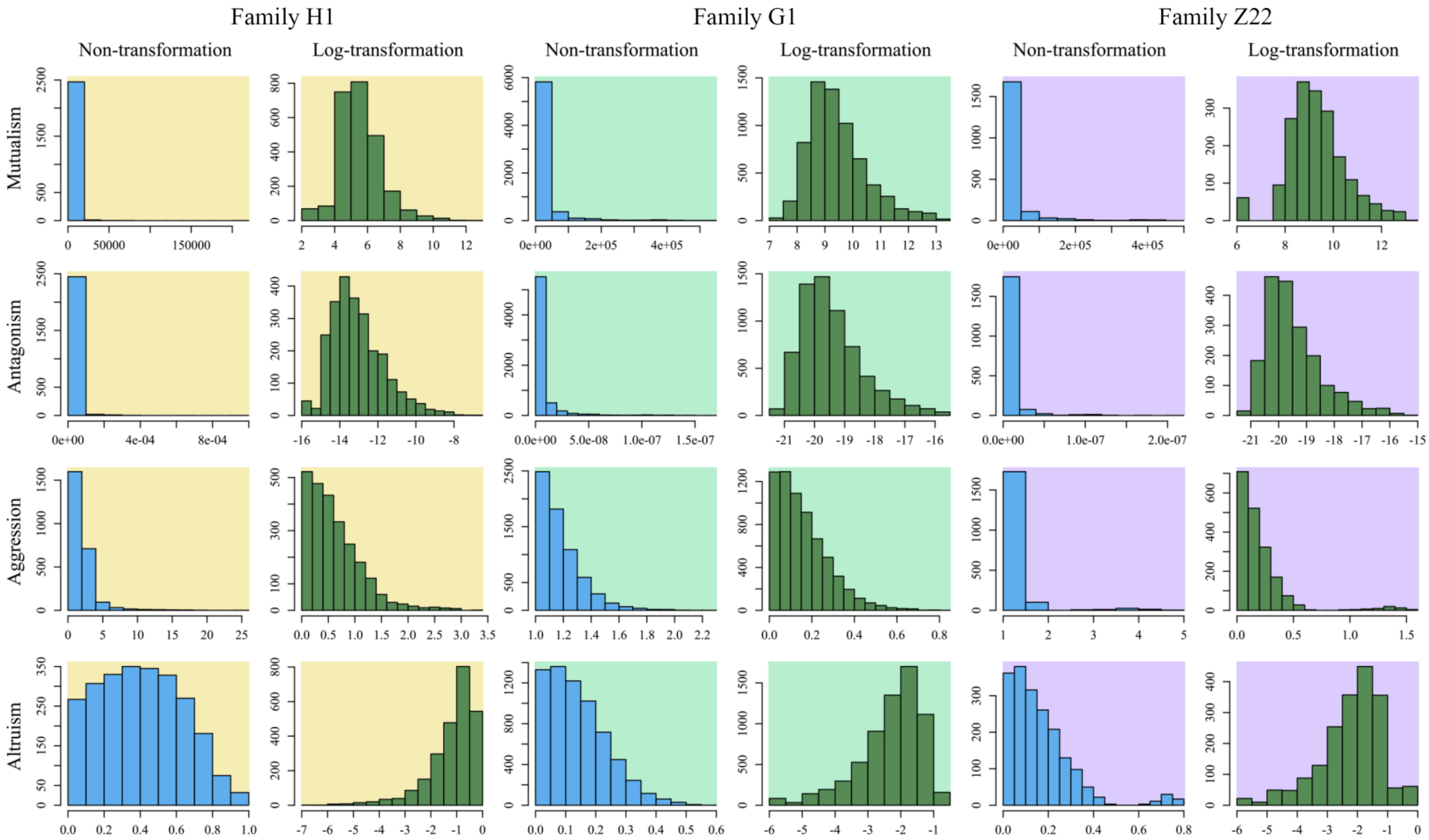

Figure S7. Histograms of mutualistic, antagonistic, aggressive, and altruistic traits, calculated from equation (1), and their log-transformation in three full-sib families, H1, G1, and Z22, of the common carp, related to Figures 4–6.

Supplementary Tables

Table S1 - S9 are excel tables.

Table S10. Proportions of the total genetic variance explained by direct genetic, indirect genetic, and genome-genome (GG) epistatic genetic effects averaged over all mutualism QTL, antagonism QTL, aggression QTL, or altruism QTL for three full-sib families of the common carp, related to Figures 5 and 6.

Family	QTL type	Testcross	Intercross	Direct	Indirect	GG epistatic
H1	Mutualism	74	23	0.532±0.077	0.285±0.075	0.183±0.080
	Antagonism	42	10	0539±0.070	0.402±0.066	0.059±0.020
	Aggression	89	25	0.515±0.079	0.252±0.075	0.233±0.105
	Altruism	70	22	0532±0.073	0.269±0.071	0.199±0.085
G1	Mutualism	40	3	0.451±0.089	0.423±0.089	0.126±0.030
	Antagonism	4	8	0.589±0.156	0.252±0.152	0.149±0.097
	Aggression	29	13	0.468±0.085	0.389±0.110	0.143±0.044
	Altruism	30	6	0.461±0.084	0.400±0.106	0.139±0.038
Z22	Mutualism	7	13	0.572±0.158	0.307±0.118	0.121±0.092
	Antagonism	4	2	0.396±0.142	0.322±0.121	0.282±0.110
	Aggression	16	13	0.519±0.132	0.379±0.131	0.102±0.084
	Altruism	21	15	0.514±0.130	0.389±0.135	0.097±0.083

Table S11. Power comparison of QTL detection from a mapping population by a traditional model and our model under different heritability (H^2) and sample sizes (m), related to Figures 4–6.

	$H^2 = 0.05$		$H^2 = 0.1$	
	$m = 70$	$m = 200$	$m = 70$	$m = 200$
Social model	0.78±0.046	0.84±0.044	0.83±0.037	0.91±0.025
Traditional model	0.31±0.094	0.34±0.073	0.35±0.028	0.38±0.031

Table S12. Toy example showing how to reformat a mapping data (left panel) into the data structure of mapping social interactions (right panel). Animals in the original data (left panel) are ordered from large to small. In each pair, a larger animal is arrayed in column L and a smaller one in column S (right panel), related to Figures 1–3. See Fig. 1 for the definition of different types of interactions.

Progeny	Marker	Phenotype
1	*AA*	w_1
2	*Aa*	w_2
3	*Aa*	w_3
4	*AA*	w_4
5	*Aa*	w_5
6	*AA*	w_6

			Social Interaction				Pair with L and S	
No.	Pair	G×G	Mutualism (z_{mu})	Antagonism (z_{an})	Aggression (z_{ag})	Altruism (z_{mu})	x|L	y\S
1	1×2	*AA*×*Aa*	$w_1w_2/(w_1-w_2)$	$1/(w_1w_2(w_1-w_2))$	w_1/w_2	$1-w_2/w_1$	w_1	w_2
2	1×3	*AA*×*Aa*	$w_1w_3/(w_1-w_3)$	$1/(w_1w_3(w_1-w_3))$	w_1/w_3	$1-w_3/w_1$	w_1	w_3
3	1×4	*AA*×*AA*	$w_1w_4/(w_1-w_4)$	$1/(w_1w_4(w_1-w_4))$	w_1/w_4	$1-w_4/w_1$	w_1	w_4
4	1×5	*AA*×*Aa*	$w_1w_5/(w_1-w_5)$	$1/(w_1w_5(w_1-w_5))$	w_1/w_5	$1-w_5/w_1$	w_1	w_5
5	1×6	*AA*×*AA*	$w_1w_6/(w_1-w_6)$	$1/(w_1w_6(w_1-w_6))$	w_1/w_6	$1-w_6/w_1$	w_1	w_6
6	2×3	*Aa*×*Aa*	$w_2w_3/(w_2-w_3)$	$1/(w_2w_3(w_2-w_3))$	w_2/w_3	$1-w_3/w_2$	w_2	w_3
7	2×4	*Aa*×*AA*	$w_2w_4/(w_2-w_4)$	$1/(w_2w_4(w_2-w_4))$	w_2/w_4	$1-w_4/w_2$	w_2	w_4
8	2×5	*Aa*×*Aa*	$w_2w_5/(w_2-w_5)$	$1/(w_2w_5(w_2-w_5))$	w_2/w_5	$1-w_5/w_2$	w_2	w_5
9	2×6	*Aa*×*AA*	$w_2w_6/(w_2-w_6)$	$1/(w_2w_6(w_2-w_6))$	w_2/w_6	$1-w_6/w_2$	w_2	w_6
10	3×4	*Aa*×*AA*	$w_3w_4/(w_3-w_4)$	$1/(w_3w_4(w_3-w_4))$	w_3/w_4	$1-w_4/w_3$	w_3	w_4
11	3×5	*Aa*×*Aa*	$w_3w_5/(w_3-w_5)$	$1/(w_3w_5(w_3-w_5))$	w_3/w_5	$1-w_5/w_3$	w_3	w_5
12	3×6	*Aa*×*AA*	$w_3w_6/(w_3-w_6)$	$1/(w_3w_6(w_3-w_6))$	w_3/w_6	$1-w_6/w_3$	w_3	w_6
13	4×5	*AA*×*Aa*	$w_4w_5/(w_4-w_5)$	$1/(w_4w_5(w_4-w_5))$	w_4/w_5	$1-w_5/w_4$	w_4	w_5
14	4×6	*AA*×*AA*	$w_4w_6/(w_4-w_6)$	$1/(w_4w_6(w_4-w_6))$	w_4/w_6	$1-w_6/w_4$	w_4	w_6
15	5×6	*Aa*×*AA*	$w_5w_6/(w_5-w_6)$	$1/(w_5w_6(w_5-w_6\|)$	w_5/w_6	$1-w_6/w_5$	w_5	w_6

Table S13. Genotypic values of four GG combinations and their underlying components: direct genetic, indirect genetic, and genome-genome (gg) epistatic effects, derived from animal pairs, each with a larger one arrayed in column L and a smaller one in column S, related to Figures 1–3.

GG Combination	Column L	Column S
Testcross Marker		
$AA \times AA$	$\mu^x_{11} = \mu_x + a_{x\leftarrow x} + a_{x\leftarrow y} + e^x_{aa}$	$\mu^y_{11} = \mu_y + a_{y\leftarrow x} + a_{y\leftarrow y} + e^y_{aa}$
$AA \times Aa$	$\mu^x_{12} = \mu_x + a_{x\leftarrow x} - a_{x\leftarrow y} - e^x_{aa}$	$\mu^y_{12} = \mu_y + a_{y\leftarrow x} - a_{y\leftarrow y} - e^y_{aa}$
$Aa \times AA$	$\mu^x_{21} = \mu_x - a_{x\leftarrow x} + a_{x\leftarrow y} - e^y_{aa}$	$\mu^y_{21} = \mu_y - a_{y\leftarrow x} + a_{y\leftarrow y} - e^y_{aa}$
$Aa \times Aa$	$\mu^x_{22} = \mu_x - a_{x\leftarrow x} - a_{x\leftarrow y} + e^x_{aa}$	$\mu^y_{22} = \mu_y - a_{y\leftarrow x} - a_{y\leftarrow y} + e^y_{aa}$
Overall Mean	$\mu_x = \frac{1}{4}(\mu^x_{11} + \mu^x_{12} + \mu^x_{21} + \mu^x_{22})$	$\mu_y = \frac{1}{4}(\mu^y_{11} + \mu^y_{12} + \mu^y_{21} + \mu^y_{22})$
Direct Effect	$a_{x\leftarrow x} = \frac{1}{4}(\mu^x_{11} + \mu^x_{12} - \mu^x_{21} - \mu^x_{22})$	$a_{y\leftarrow y} = \frac{1}{4}(\mu^y_{11} - \mu^y_{12} + \mu^y_{21} - \mu^y_{22})$
Indirect Effect	$a_{x\leftarrow y} = \frac{1}{4}(\mu^x_{11} - \mu^x_{12} + \mu^x_{21} - \mu^x_{22})$	$a_{y\leftarrow x} = \frac{1}{4}(\mu^y_{11} + \mu^y_{12} - \mu^y_{21} - \mu^y_{22})$
gg Epistasis	$e^x_{aa} = \frac{1}{4}(\mu^x_{11} - \mu^x_{12} - \mu^x_{21} + \mu^x_{22})$	$e^y_{aa} = \frac{1}{4}(\mu^y_{11} - \mu^y_{12} - \mu^y_{21} + \mu^y_{22})$
Intercross Marker		
$AA \times AA$	$\mu^x_{11} = \mu_x + a_{x\leftarrow x} + a_{x\leftarrow x} + e^x_{aa}$	$\mu^y_{11} = \mu_y + a_{y\leftarrow x} + a_{y\leftarrow y} + e^y_{aa}$
$AA \times Aa$	$\mu^x_{12} = \mu_x + a_{x\leftarrow x} + d_{x\leftarrow y} + e^x_{ad}$	$\mu^y_{12} = \mu_y + a_{y\leftarrow x} + d_{y\leftarrow y} + e^y_{ad}$
$AA \times aa$	$\mu^x_{13} = \mu_x + a_{x\leftarrow x} - a_{x\leftarrow y} - e^x_{aa}$	$\mu^y_{13} = \mu_y + a_{y\leftarrow x} - a_{x\leftarrow x} - e^x_{aa}$
$Aa \times AA$	$\mu^x_{21} = \mu_x + d_{x\leftarrow x} + a_{x\leftarrow y} + e^x_{da}$	$\mu^y_{21} = \mu_y + d_{y\leftarrow x} + a_{y\leftarrow y} + e^y_{da}$
$Aa \times Aa$	$\mu^x_{22} = \mu_x + d_{x\leftarrow x} + d_{x\leftarrow y} + e^x_{dd}$	$\mu^y_{22} = \mu_y + d_{y\leftarrow x} + d_{y\leftarrow y} + e^y_{dd}$
$Aa \times aa$	$\mu^x_{23} = \mu_x + d_{x\leftarrow x} - a_{x\leftarrow y} - e^x_{da}$	$\mu^y_{23} = \mu_y + d_{y\leftarrow x} - a_{y\leftarrow y} + e^y_{da}$
$aa \times AA$	$\mu^x_{31} = \mu_x - a_{x\leftarrow x} + a_{x\leftarrow y} - e^y_{aa}$	$\mu^y_{31} = \mu_y - a_{y\leftarrow x} + a_{y\leftarrow y} - e^y_{aa}$
$aa \times Aa$	$\mu^x_{32} = \mu_x - a_{x\leftarrow x} + d_{x\leftarrow y} - e^x_{ad}$	$\mu^y_{32} = \mu_y - a_{y\leftarrow x} + d_{y\leftarrow y} - e^y_{ad}$
$aa \times aa$	$\mu^x_{33} = \mu_x - a_{x\leftarrow x} - a_{x\leftarrow y} + e^y_{aa}$	$\mu^y_{33} = \mu_y - a_{y\leftarrow x} - a_{y\leftarrow y} + e^y_{aa}$
Overall Mean	$\mu_x = \frac{1}{9}\sum_{j_1=1}^{3}\sum_{j_2=1}^{3}\mu^x_{j_1 j_2}$	$\mu_y = \frac{1}{9}\sum_{j_1=1}^{3}\sum_{j_2=1}^{3}\mu^y_{j_1 j_2}$
Direct Effect	$a_{x\leftarrow x} = \frac{1}{4}(\mu^x_{11} + \mu^x_{13} - \mu^x_{31} - \mu^x_{33})$	$a_{y\leftarrow y} = \frac{1}{4}(\mu^y_{11} + \mu^y_{13} - \mu^y_{31} - \mu^y_{33})$
	$d_{x\leftarrow x} = \frac{1}{4}[2(\mu^x_{12} - \mu^x_{32}) - (\mu^x_{11} + \mu^x_{13} - \mu^x_{31} - \mu^x_{33})]$	$d_{y\leftarrow y} = \frac{1}{4}[2(\mu^y_{12} - \mu^y_{32}) - (\mu^y_{11} + \mu^y_{13} - \mu^y_{31} - \mu^y_{33})]$
Indirect Effect	$a_{x\leftarrow y} = \frac{1}{4}(\mu^x_{11} + \mu^x_{13} - \mu^x_{31} - \mu^x_{33})$	$a_{y\leftarrow x} = \frac{1}{4}(\mu^y_{11} + \mu^y_{13} - \mu^y_{31} - \mu^y_{33})$
	$d_{x\leftarrow y} = \frac{1}{4}[2(\mu^x_{21} - \mu^x_{23}) - (\mu^x_{11} + \mu^x_{31} - \mu^x_{13} + \mu^x_{33})]$	$d_{y\leftarrow x} = \frac{1}{4}[2(\mu^y_{21} - \mu^y_{23}) - (\mu^y_{11} + \mu^y_{31} - \mu^y_{13} - \mu^y_{33})]$
gg Epistasis	$e^x_{aa} = \frac{1}{4}(\mu^x_{11} + \mu^x_{33} - \mu^x_{13} - \mu^x_{31})$	$e^y_{aa} = \frac{1}{4}(\mu^y_{11} + \mu^y_{33} - \mu^y_{13} - \mu^y_{31})$
	$e^x_{ad} = \frac{1}{4}[2(\mu^x_{12} - \mu^x_{32}) - (\mu^x_{11} + \mu^x_{13} - \mu^x_{31} - \mu^x_{33})$	$e^y_{ad} = \frac{1}{4}[2(\mu^y_{12} - \mu^y_{32}) - (\mu^y_{11} + \mu^y_{13} - \mu^y_{31} - \mu^y_{33})$
	$e^x_{da} = \frac{1}{4}[2(\mu^x_{21} - \mu^x_{23}) - (\mu^x_{31} + \mu^x_{33} - \mu^x_{11} - \mu^x_{13})$	$e^y_{da} = \frac{1}{4}[2(\mu^y_{21} - \mu^y_{23}) - (\mu^y_{31} + \mu^y_{33} - \mu^y_{11} - \mu^y_{31})$
	$e^x_{dd} = \frac{1}{4}(2\mu^x_{22} - \mu^x_{12} - \mu^x_{21} - \mu^x_{23} - \mu^x_{32})$	$e^y_{dd} = \frac{1}{4}(2\mu^y_{22} - \mu^y_{12} - \mu^y_{21} - \mu^y_{23} - \mu^y_{32})$

Note: for a testcross QTL, $\mu^x_{j_1 j_2}$ and $\mu^y_{j_1 j_2}$ (j_1, j_2 = 1, 3) are the genotypic values of GG combinations for column A and B, respectively; $a_{x\leftarrow x}$ and $a_{y\leftarrow y}$ are the direct effects of columns A and B on their own phenotype; $a_{x\leftarrow y}$ and $a_{y\leftarrow x}$ are the indirect effects of columns B and A on the phenotype of its counterpart; and e^x_{aa} and e^y_{aa} are the genome-genome epistatic effects due to the interactions between columns A's and B's alleles on the phenotype of columns A and B, respectively. All of these definitions can be extended to an intercross QTL that includes additive (a), dominant (d), genome-genome additive × additive (e_{aa}), genome-genome additive × dominant (e_{ad}), genome-genome dominant × additive (e_{da}), and genome-genome dominant × dominant (e_{dd}) effects.

Table S14. Data structure of a toy mapping population used to infer a directed acyclic graph, related to Figures 5 and 6. Social network (**A**) was constructed from ODEs, whereas QTL networks (**B**) was constructed from dynamic Bayesian networks. σ_Q^2 is the genetic variance of a locus and μ is the population mean.

No.	QTL						Phenotype	QTL					
	Q_1	Q_2	Q_3	Q_4	Q_5	Q_6		Q_1	Q_2	Q_3	Q_4	Q_5	Q_6
S_1	*AA*	*Aa*	*AA*	*AA*	*aa*	*AA*	1.5	1.70	1.75	1.70	1.81	1.70	1.77
S_2	*aa*	*AA*	*aa*	*AA*	*Aa*	*aa*	2.12	1.94	1.94	1.86	1.81	1.88	1.98
S_3	*Aa*	*Aa*	*Aa*	*Aa*	*Aa*	*Aa*	1.85	1.89	1.75	1.87	1.86	1.88	1.81
S_4	*Aa*	*aa*	*Aa*	*aa*	*AA*	*AA*	1.91	1.89	1.84	1.87	1.81	1.88	1.77
S_5	*aa*	*AA*	*aa*	*aa*	*Aa*	*Aa*	1.76	1.94	1.94	1.86	1.81	1.88	1.81
S_6	*AA*	*aa*	*Aa*	*Aa*	*AA*	*aa*	1.84	1.70	1.84	1.87	1.86	1.88	1.98
S_7	*Aa*	*Aa*	*AA*	*Aa*	*aa*	*AA*	1.9	1.89	1.75	1.70	1.86	1.70	1.77
S_8	*AA*	*aa*	*aa*	*aa*	*Aa*	*AA*	1.77	1.70	1.84	1.86	1.81	1.88	1.77
	σ_Q^2 = 0.011	0.006	0.007	0.001	0.007	0.009	μ = 1.83	σ_Q = 0.108	0.079	0.081	0.026	0.082	0.093

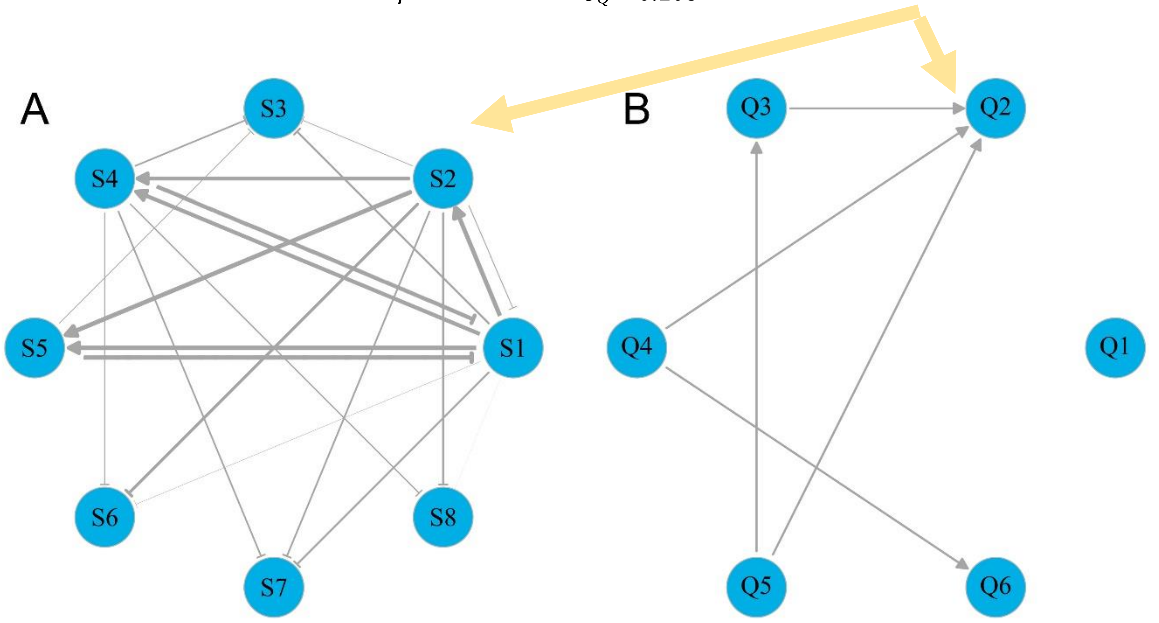

ODE-based network **Dynamic Bayesian network**

Transparent Methods

Mapping design

Consider a full-sib mapping population of animals genotyped for SNPs throughout the whole genome. By rearing them in a common environment, these animals are allowed to randomly interact with each other. The animals' resource- and space-responsive phenotypes, such as body weight and body length, are measured after the population has experienced full mutual interactions during ontogeny. To illustrate our new mapping theory, we designed a toy example in which six animals are genotyped by a testcross marker with two genotypes *AA* and *Aa* and phenotyped by a growth-related trait. Without loss of generality, we labelled and arranged the six animals in order from high to small phenotypes denoted as w_1, …, w_6 (left, **Table S12**). By pairing all animals, we reformatted genotype and phenotype data across a total of (5 × 6)/2 = 15 pairs, where a larger animal in each pair is arrayed in left column and a smaller one in right column (right, **Table S12**). Let w_L and w_S denote the phenotypic values of the larger animal L (L = 1, …, 5) and smaller animal S (S = 2, …, 6) from a pair, respectively. For a particular pair, we use the mathematic expressions, as shown in Fig. 1, to calculate the parameters z_{mu}, z_{an}, z_{ag}, and z_{al} that describe the strengths of their mutualism, antagonism, aggression, and altruism, respectively. Among all pairs, two genotypes at the marker form four possible genotype (G) × genotype (G) combinations, *AA* × *AA* (coded as 1×1), *AA* × *Aa* (coded as 1×2), *Aa* × *AA* (coded as 2×1), and *Aa* × *Aa* (coded as 2×2). A traditional mapping approach is to associate marker genotypes with trait phenotypes across individual animals (left, **Table S12**), whereas our new mapping model performs the analysis of association between GG combinations and the derived phenotypes of mutualism, antagonism, aggression, and altruism across animal-animal pairs (right, **Table S12**).

Statistical mapping of social interactions

Likelihood: We use z_i to denote the value of a derivative trait (i.e., mutualism, antagonism, aggression, or altruism) for pair i (i = 1, …, n). Let $n_{1\times1}$, $n_{1\times2}$, $n_{2\times1}$, and $n_{2\times2}$ denote the observations of GG combinations, *AA* × *AA*, *AA* × *Aa*, *Aa* × *AA*, and *Aa* × *Aa* at a test marker, respectively. The likelihood of a derivative trait at this marker is formulated as

$$L(z) = \sum_{i=1}^{n_{1\times1}} f_{1\times1}(z_i) \sum_{i=1}^{n_{1\times2}} f_{1\times2}(z_i) \sum_{i=1}^{n_{2\times1}} f_{2\times1}(z_i) \sum_{i=1}^{n_{2\times2}} f_{2\times2}(z_i) \quad (1)$$

where $f.(z_i)$ is the probability density of the derivative trait for a particular GG combination. The four derivative traits may have complicated forms of density function. However, we first test if they are normally distributed after log-transformation and implement the normal density function if they pass the test. In the subsequent data analysis of carp fish body mass, we find that all derivative traits, except for aggression, approximately follow a normal distribution after they are log-transformed (Fig. S7). The likelihood (1) has four genotypic values of GG combinations for a derivative trait, denoted as $\mu^z_{1\times1}$, $\mu^z_{1\times2}$, $\mu^z_{2\times1}$, and $\mu^z_{2\times2}$, respectively, and a residual variance. A standard likelihood approach is implemented to obtain the maximum likelihood estimates (MLEs) of these parameters.

As a ratio trait between two variables, aggression can be approached by the Cauchy density function. Let x and y denote the trait values of animal column L and S, respectively. For ratio $z_i = y_i/x_i$ ($x_i > y_i$), the density function of GG combination j_1j_2, $f_{j_1\times j_2}(z_i)$, is a product of a Cauchy density and a complicated function (Cedilnik et al., 2004), expressed as

$$f_{j_1\times j_2}(z_i)=\frac{\sigma_x\sigma_y\sqrt{1-\rho^2}}{\pi\left(\sigma_x^2z_i^2-2\rho\sigma_x\sigma_y z_i+\sigma_x^2\right)}\left[\exp(-\frac{1}{2}\sup R_{j_1\times j_2}^2)(1+\frac{R_{j_1\times j_2}\Phi\left(R_{j_1\times j_2}\right)}{\phi\left(R_{j_1\times j_2}\right)})\right]$$

$$=\frac{\sigma_x\sigma_y\sqrt{1-\rho^2}}{\pi(\sigma_y^2z_i^2-2\rho\sigma_x\sigma_y+\sigma_x^2)}\left[\exp\left(-\frac{1}{2}\sup R_{j_1\times j_2}^2\right)\right.$$

$$\left.+\sqrt{2\pi}R_{j_1\times j_2}\Phi(R_{j_1\times j_2})\exp(-\frac{1}{2}[\sup R_{j_1\times j_2}^2-R_{j_1\times j_2}^2]\right] \quad (2)$$

where

$$R_{j_1\times j_2}=\frac{\left(\sigma_y^2\mu_{j_1\times j_2}^x-\rho\sigma_x\sigma_y\mu_{j_1\times j_2}^y\right)z_i-\rho\sigma_x\sigma_y\mu_{j_1\times j_2}^x+\sigma_x^2\mu_{j_1\times j_2}^y}{\sigma_x\sigma_y\sqrt{1-\rho^2\sqrt{\sigma_y^2z_i^2-2\rho\sigma_x\sigma_y z_i+\sigma_x^2}}}$$

$$=\frac{\left(\frac{\mu_{j_1\times j_2}^x}{\sigma_x}-\rho\frac{\mu_{j_1\times j_2}^y}{\sigma_y}\right)z_i-(\rho\frac{\mu_{j_1\times j_2}^x}{\sigma_x}-\frac{\mu_{j_1\times j_2}^y}{\sigma_R})\frac{\sigma_x}{\sigma_y}}{\sqrt{1-\rho^2}\sqrt{z_i^2-2\rho\frac{\sigma_x}{\sigma_y}z_i+(\frac{\sigma_x}{\sigma_y})^2}}$$

$$\sup R_{j_1\times j_2}^2=\frac{\sigma_y^2\mu_{j_1\times j_2}^x-2\rho\sigma_x\sigma_y\mu_{j_1\times j_2}^x\mu_{j_1\times j_2}^y+\sigma_L^2\mu_{j_1\times j_2}^y}{\sigma_x^2\sigma_y^2(1-\rho)}$$

$$=\frac{(\frac{\mu_{j_1\times j_2}^x}{\sigma_x})^2-2\frac{\mu_{j_1\times j_2}^x}{\sigma_x}\frac{\mu_{j_1\times j_2}^y}{\sigma_y}+(\frac{\mu_{j_1\times j_2}^y}{\sigma_y})^2}{1-\rho^2}$$

$$\sup R_{j_1\times j_2}^2-R_{j_1\times j_2}^2=\frac{\left(\mu_{j_1\times j_2}^y-\mu_{j_1\times j_2}^x z_i\right)^2}{\sigma_x^2z_i^2-2\rho\sigma_y\sigma_x z_i+\sigma_y^2}=\frac{\left(\frac{\mu_{j_1\times j_2}^y}{\sigma_y}\frac{\sigma_y}{\sigma_x}-\frac{\mu_{j_1\times j_2}^x}{\sigma_x}z_i\right)^2}{z_i^2-2\rho\frac{\sigma_y}{\sigma_x}z_i+\left(\frac{\sigma_y}{\sigma_x}\right)^2},$$

and

$$\Phi\left(R_{j_1\times j_2}\right)=\int_o^{R_{j_1\times j_2}}\phi(r)dr=\int_o^{R_{j_1\times j_2}}\frac{1}{\sqrt{2\pi}}e^{-\frac{r^2}{2}}dr=\frac{1}{2}erf(\frac{R_{j_1\times j_2}}{\sqrt{2}})$$

is the error function. The density function (2) was implemented into the likelihood (1) that is defined by GG combination-dependent genotypic means of variables *x* ($\mu_{j_1\times j_2}^x$) and *y* ($\mu_{j_1\times j_2}^y$), the residual variances of variables *x* (σ_x^2) and *y* (σ_y^2), and the correlation between the two variables (ρ).

Significance test: To determine whether a significant QTL exists to affect a type of social interaction, we compare the genotypic difference among GG combinations. For mutualism, antagonism, and altruism traits, four genotypic values of the derivative trait are estimated directly. For the aggression trait, its genotypic values are estimated as $\mu_{j_1\times j_2}^z=\mu_{j_1\times j_2}^y/\mu_{j_1\times j_2}^x$. In general, we formulate the following hypotheses for significance test:

H_0: $\mu_{1\times1}^z=\mu_{1\times2}^z=\mu_{2\times1}^z=\mu_{2\times2}^z=\mu^z$

H_1: At least one of the above equalities does not hold. (3)

A log-likelihood ratio calculated from the H_0 (there is no QTL) and H_1 hypothesis (there is a QTL) is used to test if these GG combinations differ from each other for a derivative trait. If the null hypothesis is rejected, then we could claim the existence of a significant QTL that

affects the derivative trait. We call such a QTL a mutualism QTL, antagonism QTL, aggression QTL, or altruism QTL if the derivative trait is z_{mu}, z_{an}, z_{ag}, or z_{al}, respectively. The critical threshold for the significance test can be empirically determined through permutation tests.

Quantitative genetic dissection of social interactions

After a significant QTL for an interaction parameter is detected, the new theory can be used to test how this QTL affects phenotypic variation. As described above, each pair is composed of two animals, a larger one L arrayed in left column (with trait value denoted as x) and a smaller one S in right column (with trait value denoted as y) (right, **Table S12**). For a QTL significant by test, we calculate the MLEs of the genotypic value of each GG combination for each column, i.e., $\mu^x_{j_1 j_2}$ for column L and $\mu^y_{j_1 j_2}$ for column S (j_1, j_2 = 1 for AA, 2 for Aa). According to quantitative genetic theory, we partitioned these genotypic values into their underlying components (**Table S13**), including the overall means for different columns, denoted as μ_x for column L and μ_y for column S; direct genetic effects of QTL alleles from two columns on their own phenotypes, denoted as $a_{x\leftarrow x}$ for column L and $a_{y\leftarrow y}$ for column S; indirect genetic effects of QTL alleles from two columns on each other's phenotypes, denoted as $a_{x\leftarrow y}$ for column S affecting column L and $a_{y\leftarrow x}$ for column L affecting column S; and genome-genome epistatic effects of QTL alleles from different columns, denoted as e^x_{aa} on column L and e^y_{aa} on column S.

Based on the component structure of a GG combination genotypic value (**Table S13**), we solve these effect parameters by

$$\begin{bmatrix} a_{x\leftarrow x} \\ a_{x\leftarrow y} \\ e^x_{aa} \end{bmatrix} = \frac{1}{4}\begin{bmatrix} 1 & 1 & -1 & -1 \\ 1 & -1 & 1 & -1 \\ 1 & -1 & -1 & 1 \end{bmatrix}\begin{bmatrix} \mu^x_{11} \\ \mu^x_{12} \\ \mu^x_{21} \\ \mu^x_{22} \end{bmatrix}, \begin{bmatrix} a_{y\leftarrow x} \\ a_{y\leftarrow y} \\ e^y_{aa} \end{bmatrix} = \frac{1}{4}\begin{bmatrix} 1 & 1 & -1 & -1 \\ 1 & -1 & 1 & -1 \\ 1 & -1 & -1 & 1 \end{bmatrix}\begin{bmatrix} \mu^y_{11} \\ \mu^y_{12} \\ \mu^y_{21} \\ \mu^y_{22} \end{bmatrix} \quad (4)$$

After these effect parameters are estimated, we formulate a procedure to test the significance of each of them by a log-likelihood ratio approach. For example, the null hypotheses for testing these effects are expressed as

$$H_0: a_{x\leftarrow x} = a_{y\leftarrow y} = 0, \text{ for the direct effect} \quad (5)$$

$$H_0: a_{x\leftarrow y} = a_{y\leftarrow x} = 0, \text{ for the indirect effect} \quad (6)$$

$$H_0: e^x_{aa} = e^y_{aa} = 0, \text{ for the fish-fish epistatic effect} \quad (7)$$

The critical thresholds for all the above hypotheses tests can be obtained from classic chi-square statistics or simulation studies. If these effects are significant, we calculate their means over two columns, i.e., $a_D = (a_{x\leftarrow x} + a_{y\leftarrow y})/2$, $a_I = (a_{x\leftarrow y} + a_{y\leftarrow x})/2$, and $e_{aa} = (e^x_{aa} + e^y_{aa})/2$, as the estimates of direct, indirect, and genome-genome epistatic effects on a phenotypic trait in the population.

We next describe a procedure to estimate genetic variances due to direct, indirect, and genome-genome epistatic effects. Considering columns L and S of the right part of **Table S12**, we calculate the genetic variance among the four GG combinations at a significant QTL, denoted as V^x_G for column L and V^y_G for column S. The mean of V^x_G and V^y_G, expressed as V_G, is the estimation of the total genetic variance explained by the QTL. Based on column L's and S's genotypes, we calculate the genetic variance of the trait in columns L and S, respectively, denoted as $V_{x\leftarrow x}$ for column L and $V_{y\leftarrow y}$ for column S, whose mean is the estimated direct genetic variance of the QTL, expressed as V_D. Similarly, using column L's and S's genotypes, we can calculate the genetic variance of the trait in columns S and L, respectively, denoted as $V_{y\leftarrow x}$ for column L affecting S and $V_{x\leftarrow y}$ for column S affecting L. The mean of these two genetic

variances, expressed as V_I, is the estimated indirect genetic variance of the QTL. For columns L and S, we calculate $V_{aa}^{x} = V_G^x - V_{x \leftarrow x} - V_{x \leftarrow y}$ and $V_{aa}^{y} = V_G^y - V_{y \leftarrow y} - V_{y \leftarrow x}$, respectively, and their mean is the genome-genome epistatic genetic variance of the QTL, denoted as V_{aa}. We can further calculate the proportions of direct, indirect, and genome-genome epistatic effects to the total genetic variance by this QTL.

For an outcrossing species like the carp fish, a full-sib family population derived from two heterozygous parents may include two types of markers, i.e., testcross markers at which one parent is heterozygous whereas the other is homozygous, and intercross markers at which both parents are heterozygous (Wu et al., 2002; Lu et al., 2004). The procedure described above can be similarly used to map mutualism, antagonism, aggression, or altruism QTL based on intercross markers. For an intercross marker with three genotypes (*AA*, *Aa*, and *aa)*, we use nine GG combinations, $AA \times AA$ (coded as 1×1), $AA \times Aa$ (coded as 1×2), $AA \times aa$ (coded as 1×3), $Aa \times AA$ (coded as 2×1), $Aa \times Aa$ (coded as 2×2), $Aa \times aa$ (coded as 2×3), $aa \times Aa$ (coded as 3×1), $aa \times Aa$ (coded as 3×2), and $aa \times aa$ (coded as 3×3). Similarly, we formulated a log-likelihood approach to estimate the MLEs of genotypic values of nine GG combinations for columns L and S, and tested and estimated the significance of direct additive (a_D) and dominant genetic effects (d_D), indirect additive (a_I), and dominant genetic effects (d_I), and genome-genome additive-additive (e_{AA}), genome-genome additive-dominant (e_{AD}), genome-genome dominant-additive (e_{DA}), and genome-genome dominant-dominant epistatic genetic effects (e_{DD}) (**Table S13**).

Inferring directed acyclic networks

To better understand how different types of QTL, mutualistic, antagonistic, aggressive, or altruistic, jointly affect the phenotypic trait of animals, we develop and implement a statistical algorithm to infer a directed acyclic graph (DAG) of QTL interactions. We use a toy example to explain our algorithm. Suppose there are six QTL under consideration, each with three genotypes *AA* (coded as 1), *Aa* (coded as 2) and *aa* (coded as 3). These QTL are segregating in the mapping population of eight phenotyped animals (left, **Table S14**). For the trait measured, we calculate its population mean (μ) averaged over all animals and also its marginal genotypic means μ_{jk} (j = 1, 2, 3) over the animals carrying the same genotype at each QTL k. Now, we assign each genotype at each QTL by its marginal genotypic mean to form an (8 × 6) matrix of genotypic values (right, **Table S14**), from which two types of DAG, constructed by different QTL and different animals, respectively, were inferred by Bayesian networks.

QTL network: The structure of a QTL network is defined by two sets: the set of nodes (vertices) represented by individual QTL and the set of directed edges of dependence (directed epistasis) among the QTL. Because each QTL has three distinct genotypes, its marginal genotypic values (right, **Table S14**) can be better viewed as ordinal variables. The most general approach for constructing ordinal Bayesian networks is to treat ordinal variables as nominal so that nominal techniques can be used. However, this treatment entails a loss of information because the ordering among categories is not considered. At present, only a few ordinal-sensitive procedures for learning Bayesian network from ordinal data have been developed in order to preserve the ordering of ordinal data (Musella, 2013).

Following Musella's procedure (Musella, 2013), we describe an ordinal PC algorithm for learning and inferring a QTL DAG from marginal ranked genotypic values. Consider data structure, given in **Table S14** (right), composed of eight samples on six QTL variables. The PC algorithm is a stepwise backward algorithm for DAG inference (Spirtes et al., 2013). We

first tested the conditional independence $Q_1 \perp Q_3 | Q_2$ where Q_1, Q_2 and Q_3 are ordinal, each with three genotypes. We let $n_{j_1 j_2 j_3}$ denote the observation of the j_1-th genotype of Q_1 (j_1 = 1, 2, 3), j_2-th genotype of Q_2 (j_1 = 1, 2, 3), and j_3-th genotype of Q_3 (j_1 = 1, 2, 3). Let $F_{j_1 j_2}(Q_3)$ denote the conditional distribution of Q_3 given $Q_1 = j_1$ and $Q_2 = j_2$. The null hypothesis of the test is formulated as

$$\text{H}_0\text{: } F_{1j_2}(j_3) = F_{2j_2}(j_3) = F_{3j_2}(j_3), \forall j_2, \forall j_3 \quad (8)$$

whose alternative test reflecting a stochastic ordering among distributions is written as

$$\text{H}_1\text{:} \begin{cases} F_{j_1 j_2}(j_3) > F_{j'_1 j_2}(j_3) \\ F_{j_1 j_2}(j_3) < F_{j'_1 j_2}(j_3) \end{cases}, \text{ with } j_1 < j'_1, \forall j_2, \forall j_3 \quad (9)$$

From tests (8) and (9), we calculate a so-called Jonkheere-Terpstra (JT) test statistic as

$$\text{JT} = \sum_{j_2=1}^{3} \sum_{j_1=1}^{3} \sum_{j'_1=1}^{j_1-1} \left[\sum_{s=1}^{3} w_{j_1 j'_1 s j_2} n_{j_1 s j_2} - \frac{n_{j_1 \cdot j_2}(n_{j_1 \cdot j_2} + 1)}{2} \right] \quad (10)$$

where $w_{j_1 j'_1 s j_2}$ was the Wilcoxon score described by

$$w_{j_1 j'_1 s j_2} = \sum_{t=1}^{s-1} (n_{j_1 t j_2} + n_{j'_1 t j_2}) + \frac{n_{j_1 s j_2} + n_{j'_1 s j_2} + 1}{2}. \quad (11)$$

Under the null hypothesis, the mean of JT was calculated as

$$E(\text{JT}|\text{H}_0) = \frac{1}{4} \sum_{j_2=1}^{3} (n_{\cdot\cdot j_2}^2 - \sum_{j_1=1}^{3} n_{j_1 \cdot j_2}^2)$$

.

Per Lehmann (D'Abrera and Lehmann, 1975) and Pirie (Pirie, 1983), we derive the asymptotic variance of JT under the null hypothesis as

$$\widehat{Var}(\text{JT}|\text{H}_0) = \frac{V_1}{72} + \frac{V_2}{36(n_{\cdot\cdot j_2} - 1)(n_{\cdot\cdot j_2} - 2)} + \frac{V_3}{8(n_{\cdot\cdot j_2}(n_{\cdot\cdot j_2} - 1))}$$

where

$V_1 = n_{\cdot\cdot j_2}(n_{\cdot\cdot j_2} - 1)(2n_{\cdot\cdot j_2} + 5) - \sum_{j_3}^{3}(n_{j_1 \cdot j_2}(n_{j_1 \cdot j_2} + 5) - \sum_{j_3}^{3}(n_{\cdot j_2 j_3} - 1)(2n_{\cdot j_2 j_3} + 5)),$
$V_2 = \sum_{j_1}^{3}(n_{j_1 \cdot j_2}(n_{j_1 \cdot j_2} - 1)(2n_{j_1 \cdot j_2} - 2) - \sum_{j_3}^{3}(n_{\cdot j_2 j_3} - 1)(n_{\cdot j_2 j_3} - 2)),$
$V_3 = \sum_{j_1}^{3}(n_{j_1 \cdot j_2}(n_{j_1 \cdot j_2} - 1)) - \sum_{j_3}^{3}(n_{\cdot j_2 j_3}(n_{\cdot j_2 j_3} - 1)).$

Based on these derivations, it can be proved that the test statistic is asymptotically normally distributed.

The ordinal PC algorithm infers a QTL DAG (Musella, 2013) using the three steps as follows:

Step 1: Build the skeleton of the graph. Starting with a complete undirected graph, we obtain a graph where all QTL are connected to each other. Given a chosen significance level, statistical tests based on the Jonkheere-Terpstra test are performed to decide if we need to remove or maintain edges between QTL in the graph. This procedure leads to the detection of the skeleton of the graph.

Step 2: Find v-configurations. If two QTL, Q_1 and Q_2, are not conditionally independent given a QTL Q_3, then Q_3 is a collider node or a common sink and a v-configuration $Q_1 \rightarrow Q_3 \leftarrow Q_2$ (i.e., converging directed edges into the same node) is drawn; otherwise edges remain undirected $Q_1 - Q_3 - Q_2$.

Step 3: Create no new v-configuration. Some constraints must be given to orient other edges without creating additional colliders or some cycles.

Musella (2013) showed that the ordinal PC algorithm outperforms the PC algorithm (for modeling discrete data without considering their ranking) in terms of sensitivity (specified by true positive rate) and precision (specified by true discovery rate), especially when sample size is small. Yet, the two algorithms do not differ dramatically in specificity (specified by false positive rate).

Social network: Different animals interact with each other through mutualism, antagonism, aggression, or altruism to form a community. We implement an ordinary differential equation (ODE) approach proposed by Wu et al. (2014) to investigate how QTL modulate the structure and organization of an animal-animal interaction network. In such a QTL-driven social network, the nodes are individual animals and the edges are animal-animal interactions whose direction, sign, and strength are determined by QTL. Consider **Table S14**'s toy example for a mapping population, where eight animals were each collected by six markers. We assign each individual at a given QTL by a value, i.e., its marginal genotypic mean at this QTL. Taken together, we obtain an (8×6) matrix of genotypic values. Unlike a QTL each with three ranked categories of genotypic values over all animals, each animal form a set of somewhat continuous genotypic values across QTL (right, **Table S14**). Next, we show that a nonparametric approach can be used to model how an animal changes its genotypic value over QTL.

Let g_{ik} denote the genotypic value of individual i ($i = 1, \ldots, 8$) at QTL k ($k = 1, \ldots, 6$). Note that g_{ik} depends on the genotype individual i carries at QTL k. Because of social interactions, the genotypic value of one individual is affected by or affects those of other individuals. This allows us to formulate a system of ODEs, expressed as

$$\dot{g}_{ik} = f_i(g_{ik}) + \sum_{i \neq i',\, i'=1}^{8} h_{i \leftarrow i'}\left(g_{i'k}\right) \tag{12}$$

where $\dot{g}_{ik}$ is the rate of the overall change of genotypic value for individual i from one QTL to next, $f_i(g_{ik})$ is the function that describes the change rate of individual i's genotypic value independent of any other individuals, and $h_{i\leftarrow i'}\left(g_{i'k}\right)$ is the function that specifies the change rate of genotypic value due to the impact of any other individual i' that affects individual i. $h_{i\leftarrow i'}\left(g_{i'k}\right)$ determines the sign, direction, and strength of social interaction between individual i and i'. If both $h_{i\leftarrow i'}\left(g_{i'k}\right)$ and $h_{i'\leftarrow i}(g_{ik})$ are positive or negative, this suggests that these two individuals are mutualistic and antagonistic, respectively. If both are zero, then the two individuals have no interactions. If $h_{i\leftarrow i'}\left(g_{i'k}\right)$ is positive or zero but $h_{i'\leftarrow i}(g_{ik})$ is negative, this indicates that individual i is aggressive on individual i'. If $h_{i\leftarrow i'}\left(g_{i'k}\right)$ is positive but $h_{i'\leftarrow i}(g_{ik})$ is zero, this shows that individual i' is altruistic for individual i. The magnitudes of $h_{i\leftarrow i'}\left(g_{i'k}\right)$ and $h_{i'\leftarrow i}(g_{ik})$ can quantify the strength of social interactions.

To solve ODE (12), we integrate three rules from different disciplines. First, in sociological studies, there is the Dunbar's law, stating that the number of stable relationships a human can comfortably maintain in his social network is not beyond a limit (Dunbar, 1992). Under the Dunbar's law, using real data from both hunter-gather and modern communities, Harre and Prokopenko (2016) calculated the average number of links maintained by individuals to form cooperative groups, which changes with group size. For example, a person in a group of five,

15, 45, and 132 needs to maintain an average of one to two, two to three, three to four links, and four to five links respectively. This rule can largely simplify our joint modeling of ODEs (12), making ODE parameter estimation and interpretation feasible and tractable, especially when the dimension of this equation group is large. Second, statistical rules of variable selection can be used to determine an optimal small set of individuals that interact stably with a focal individual. Group LASSO (Yuan and Lin, 2006) and adaptive group LASSO (Wang and Leng, 2008) derived from Tibshirani's original LASSO (Tibshirani, 1996) have proven to be powerful for variable selection. In previous studies, several authors have already worked out model selection in high-dimensional ODEs (Lu et al., 2011; Henderson and Michailidis, 2014; Wu et al., 2014). Thus, the integration of the Dunbar's law with variable selection enables the construction of a sparse social network, which facilitates the characterization of most important social connections within the network.

Third, how does the locus-varying genotypic value of an individual change from one QTL to next? In quantitative genetics, the genetic effect of a QTL is generally positively associated with its genetic variance. Based on this rule, we can model the genotypic value of an individual at a QTL as a function of standard genetic deviation explained by this QTL. By arranging all QTL in an order of their genetic deviations, we incorporate a Legendre Orthogonal Polynomial (LOP)-based nonparametric approach to fit the functions, $f_i(g_{ik})$ and $h_{i\leftarrow i'}\left(g_{i'k}\right)$, that jointly describe QTL-varying genotypic value of each individual in equation (12). Because of its advantage in orthogonality and efficient convergence, the LOP is effective for modeling the curves of any complex form using sparse data in quantitative genetic studies (Das et al., 2011; Jiang et al., 2016). The LOP, a solution of the Legendre differential equation,

$$(1-v^2)\frac{d^2u}{dv^2}-2v\frac{du}{dv}+r(r+1)u=0$$

can be expressed as

$$P_r(v)=\sum_{c=0}^{C}(-1)^c\frac{(2r-2c)!}{2^r!\,(r-c)!\,(r-2c)!}v^{r-2c}$$

which is called the Legendre polynomial of order r, where C is an integer, expressed as $r/2$ or $(r-1)/2$, and v is an independent variable, i.e., standard genetic deviation in social network modeling. In practice, it needs to be corrected as $t^* = 1 + 2(t - t_{\max})/(t_{\max} - t_{\min})$ within interval [–1,1], where $t_{\min}$ and $t_{\max}$ are the two extreme points at the low and high end, respectively. By defining a series of basis values, the LOP is used to determine the curvature of QTL-varying genotypic values by choosing an optimal polynomial order.

In **Table S14** (right), we construct the QTL network and social network for the toy example by the above approaches. These toy networks can help the readers better understand the utility of these approaches.

Monte Carol simulation

To examine the statistical properties of the new model, we perform computer simulation by mimicking the data structure of a mapping population. We show how to simulate the phenotypic data of a trait under the constraint of animal-animal interactions. We let w_{j_1} and w_{j_2} denote the phenotypic value of animal i_1 and i_2 ($0 \le i_1 < i_2 \le m$), respectively, from the population of m animals and z_i ($i = 1, \ldots, m(m-1)/2$) denote the strength of one of their interaction types, as defined in Fig. 1. To simulate the trait data of the pairing animals, we need to determine the variance of phenotypic values among m animals under the constraint of a social interaction considered. This requires us to derive the variance of an interaction derivative

(z_i) among $m(m–1)/2$ pairs in terms of the variances of w_{j_1} and w_{j_2}. As an example, we assume that the aggression variable, expressed as the ratio of a larger to smaller animal, is simulated.

We reformat the data by adding reciprocal pairs, generating a total of $m(m–1)$ pairs. Let $x > y$ denote the phenotypic values of animals at the left and right sides, respectively, in pairs. Assuming that x and y are independent, the variance of $z = x/y$ is expressed by

$$V_Z = \frac{1}{\bar{y}^4}(\bar{y}^2 V_X + \bar{x}^2 V_Y - 2\rho\bar{x}\bar{y}\sqrt{V_X V_Y}, \quad (13)$$

where $\bar{x}$ and $\bar{y}$ are the means of x and y variables, V_X and V_Y are the variances of x and y variables, and ρ is the correlation between the two variables, respectively. In spite of different orders, x and y variables contained the same set of phenotypic data for m fish so that $V_X = V_Y$ and $\bar{x} = \bar{y}$.

Consider a testcross QTL with two genotypes *AA* and *Aa* of an equal proportion in the mapping population. A total of $m(m–1)$ pairs are clustered into four GG combinations (right panel, **Table S12**). The phenotypic data of an animal pair are simulated by summing the GG genotypic value and a residual error with mean zero and residual variance (V_Z) (14) scaled by the heritability (H_Z^2) explained by GG combinations at the assumed QTL. From the simulated data of animal pairs, we need to simulate the phenotypic data of individual animals (left, **Table S12**). This was done by randomly sampling values of m animals that meet their ratios across $m(m–1)/2$ pairs, but under the constraint that m animals' values have a variance $\sqrt{V_Z}$, expressed by equation (13).

Our simulation was based on four scenarios designed per GG heritability H_Z^2 (large 0.10 vs. small 0.05) and mapping size m (large 200 vs. small 70). For each scenario, we calculated the accuracy and precision of each genetic effect (such as the direct, indirect, and genome-genome epistatic effects) from pairwise data (right, **Table S12**) by the new model, testing the statistical property of the new model. Also, we calculate and compare the power of QTL detection by the new model and the traditional model that analyzes original data directly (left, **Table S12**). The false positive rates of the new model is also calculated.

Mapping experiment

Animal material: The experiment of genetic mapping was conducted using an F_1 family (named H1) of *Cyprinus carpio* including 71 progeny produced by Hebao Red carp and Koi carp. The fish were cultured at the Research Institute for Heilongjiang River Fisheries, Harbin, China, and measured for body mass after anesthesia with MS222 when they reached an adult stage of fish growth. The H1 family was served as the discovery cohort, whose results were directly used to test our new theory. To validate the results discovered by the new theory, we replicated the mapping study by two additional F_1 families (named G1 and Z22) of Yellow River carp. These two families with 115 and 62 progeny, respectively, were cultured at the Henan Academy of Fishery Sciences, Zhengzhou, China. The same trait, body mass, was measured for each family at the adult stage of fish growth.

SNP array genotyping and quality control: Genomic DNA was extracted from blood samples (400–800μl caudal peduncle) of the hybrids and their parents using a DNeasy Blood & Tissue Kit (Qiagen, Shanghai, China) following manufacturer's protocol. DNA was quantified by Nanovue (Thermo Scientific) and the integrity of DNA was examined by 1% agarose gel electrophoresis stained with ethidium bromide. Then qualified DNA was

genotyped using the Affymetrix Axiom Carp SNP array containing ~250 K validated SNPs (P. Xu et al., 2014; J. Xu et al., 2014). Genotyping results were provided by GeneSeek (Lincoln, Nebraska, USA). After quality control, we obtained 39,960 Mendelian segregating SNPs throughout the common carp genome of size ~1.42 Gb in the H1 family, from which a high-density linkage map, with an average marker interval of 0.75 cM, was constructed by OneMap (Margarido et al., 2007). For G1 and Z22 families, we genotyped 39,960, 97,532, and 86,370 SNPs following Mendel's first law, respectively.

Annotation of candidate genes: The significant SNPs detected by our theory are annotated using the database of the common carp genome project. Genes located within upstream and downstream 10 kb of the candidate SNPs were selected. Based on the selected gene lists, Gene Ontology and KEGG Enrichment analyses are conducted using DAVID (Huang et al., 2009; Huang et al., 2009) online programs with default parameters, and figures are drawn using REVIGO software (Supek et al., 2011).

References

Cedilnik, A., Kosmelj, K. and Blejec, A. (2004). The distribution of the ratio of jointly normal variables. *Metodol Zvezki* 1, 99-108.

Das, K., Li, J.H., Wang, Z., Fu, G., Li, Y., Mauger, D., Li, R. and Wu, R.L. (2011). A dynamic model for genome-wide association studies. *Human Genetics* 129, 629–639.

Dunbar, R.I.M. (1993). Neocortex size as a constraint on group size in primates. *Journal of Human Evolution* 22, 469–493.

Harre, M. and Prokopenko, M. (2016). The social brain: Scale-invariant layering of Erdos-Renyi networks in small-scale human societies. *Journal of the Royal Society Interface* 13, 1-6.

Henderson, J. and Michailidis, G. (2014). Network reconstruction using nonparametric additive ODE models. *PLoS ONE* 9(4), e94003.

Huang, D. W., Sherman, B. T. and Lempicki, R. A. (2009). Bioinformatics enrichment tools: paths toward the comprehensive functional analysis of large gene lists. *Nucleic Acids Research* 37, 1–13.

Huang, D. W. , Sherman, B. T. and Lempicki, R. A. (2009). Systematic and integrative analysis of large gene lists using DAVID bioinformatics resources. *Nature Protocols* 4, 44–57.

Jiang, L.B., Ye, M.X., Zhu, X.L., Sang, M.M. and Wu, R.L. (2016). Evo-Devo-Epi*R*: A genome-wide search platform for epistatic control on the evolution of development. *Briefings in Bioinformatics* 18, 754-760.

D'Abrera, H.J.M. and Lehmann, E.I. (1975). Nonparametrics: Statistical Methods Based on Ranks. Holden-Day.

Lu, T., Liang, H., Li, H. and Wu, H. (2011). High-dimensional ODEs coupled with mixed-effects modeling techniques for dynamic gene regulatory network identification. Journal of American Statistical Association 106, 1242–1258.

Lu, Q., Cui, Y.H. and Wu, R.L. (2004). A multilocus likelihood approach to joint modeling of linkage, parental diplotype and gene order in a full-sib family. *BMC Genetics* 5, 20.

Margarido, G.R.A., Souza, A.P. and Garcia, A.A.F. (2007). OneMap: Software for genetic mapping in outcrossing species. *Hereditas* 144, 78–79.

Musella, F. (2013). A PC algorithm variation for ordinal variables. *Computational Statistics* 28, 2749–2759.

Pirie, W. (1983). Jonckheere tests for ordered alternatives. *Encyclopedia of Statistical Science* 4, 315–318.

Spirtes, P., Glymour, C. and Scheines, R. (2000). Causation, Prediction, and Search, 2nd edn, MIT Press.

Supek, F., Bošnjak, M., Škunca, N. and Šmuc, T. (2011). REVIGO summarizes and visualizes long lists of gene ontology terms. *PLoS One* 6(7), e218000.
Tibshirani, R.J. (1996). Regression shrinkage and selection via the LASSO. *Journal of Royal Statistical Society B* 58, 267-288.
Wang, H. and Leng, C. (2008). A note on adaptive group LASSO. *Computational Statistics and Data Analysis* 52, 5277-5286.
Wu, H., Lu, T., Xue, H. and Liang, H. (2014). Sparse additive ordinary differential equations for dynamic gene regulatory network modeling. *Journal of American Statistical Association* 109, 700-716.
Wu, R., Ma, C., Painter, I. and Zeng, Z. (2002). Simultaneous maximum likelihood estimation of linkage and linkage phases in outcrossing species. *Theoretical Population Biology* 61, 349–363.
Xu, J., Zhao, Z., Zhang, X., Zheng, X., Li, J., Jiang, Y., Kuang, Y., Zhang, Y. et al. (2014). Development and evaluation of the first high-throughput SNP array for common carp (*Cyprinus carpio*). *BMC Genomics* 15: 307.
Xu, P., Zhang, X., Wang, X., Li, J., Liu, G., Kuang, Y., Xu, J., Zheng, X., Ren, L., Wang, G., Zhang, Y. and Zhang, Y. (2014). Genome sequence and genetic diversity of the common carp. *Nature Genetics* 46, 1212–1219.
Yuan, M. and Lin, Y. (2006). Model selection and estimation in regression with grouped variables. *Journal of Royal Statistical Society B* 68, 49-67.
Zhu, X., Jiang, L., Ye, M., Sun, L., Gragnoli, C. and Wu, R.L. (2016). Integrating evolutionary game theory into mechanistic genotype-phenotype mapping. *Trends in Genetics* 32, 256-268.
